# Early detection and progression of insulin resistance revealed by impaired organismal anti-inflammatory heat shock response during *ex vivo* whole-blood heat challenge

**DOI:** 10.1042/CS20243515

**Published:** 2025-01-23

**Authors:** Helena Trevisan Schroeder, Carlos Henrique de Lemos Muller, Maria Inês Lavina Rodrigues, Marcela Alves de Azevedo, Thiago Gomes Heck, Mauricio Krause, Paulo Ivo Homem de Bittencourt Jr.

**Affiliations:** 1 Laboratory of Cellular Physiology (FisCel), Department of Physiology, Institute of Basic Health Sciences (ICBS), Federal University of Rio Grande do Sul (UFRGS), Rua Ramiro Barcelos 2600, laboratory 646, 90035-003 Porto Alegre, RS, Brazil; 2 Laboratory of Inflammation, Metabolism and Exercise Research (LAPIMEX), Department of Physiology, ICBS, UFRGS, 90035-003 Porto Alegre, RS, Brazil; 3 Postgraduate Program in Integral Health Care (PPGAIS-UNIJUÍ/UNICRUZ/URI), Regional University of Northwestern Rio Grande Do Sul State (UNIJUI), 98700-000 Ijuí, RS, Brazil; 4 Postgraduate Program in Mathematical and Computational Modelling (PPGMMC), UNIJUI, 98700-000 Ijuí, RS, Brazil

**Keywords:** heat shock response, high-fat diet, HSP70, insulin resistance, low-grade inflammation, obesity, type-2 diabetes

## Abstract

Chronic inflammatory diseases, e.g., obesity, cardiovascular disease and type-2 diabetes, progressively suppress the anti-inflammatory heat shock response (HSR) by impairing the synthesis of key components, perpetuating inflammation. Monitoring HSR progression offers predictive value for countering chronic inflammation. This study quantified HSR in high-fat diet (HFD) and normal chow (NC) mice by measuring 70 kDa heat shock protein (HSP70) expression after heat treatment of whole blood samples. To align with human translational relevance, animals were housed within their thermoneutral zone (TNZ). Whole blood was heat-challenged weekly at 42 °C for 1–2 hours over 22 weeks, and ΔHSP70 was calculated as the difference between HSP70 expressions at 42 °C and 37 °C. Results correlated with fasting glycaemia, oral glucose tolerance test, intraperitoneal insulin tolerance test and 2-hour post-glucose load glycaemia. ΔHSP70 levels >0.2250 indicated normal fasting glycaemia, while levels <0.2125 signalled insulin resistance and type-2 diabetes onset. A logistic model (five-parameter logistic) showed progressive HSR decline, with HFD mice exhibiting earlier ΔHSP70 reduction (*t*_1/2_ = 3.14 weeks) compared with NC mice (*t*_1/2_ = 8.24 weeks), highlighting compromised anti-inflammatory capacity in both groups of mice maintained at TNZ. Remarkably, even NC mice surpassed insulin resistance thresholds by week 22, relevant as control diets confronted interventions. Observed HSR decline mirrors tissue-level suppression in obese and type-2 diabetic individuals, underscoring HSR failure as a hallmark of obesity-driven inflammation. This study introduces a practical whole-blood assay to evaluate HSR suppression, allowing assessment of glycaemic status during obesity onset before any clinical manifestation.

## Introduction

The heat shock response (HSR) is a highly evolutionarily conserved anti-inflammatory biochemical pathway primarily dedicated to maintaining cellular proteostasis and metabolic equilibrium while mitigating inflammation. The intricate mechanisms involved in the HSR orchestrate a harmonious response to diverse challenges, including exercise, endoplasmic reticulum (ER) stress, positive energy imbalances and other homeostasis-threatening situations (e.g., thermal, oxidative and low-energy metabolic stresses) that are coupled to the resolution of inflammation.

Through the production of anti-aggregative heat shock proteins (HSPs), the HSR actively resolves inflammation and maintains the body’s delicate immunoinflammatory balance to prevent immunosuppression or excessive inflammation [1–3]. This equilibrium, now termed ‘caloristasis’ [4,5], ensures that cellular homeostasis remains intact despite fluctuating internal and external conditions. Regrettably, chronic degenerative diseases with an inflammatory component are all linked to a gradual decline in HSR effectiveness, hindering the physiological resolution of inflammation and leading to chronic low-grade inflammation.

The core of the HSR revolves around the 70 kDa family of HSPs (HSP70) and other molecular chaperones triggered by heat shock factor-1 (HSF1) activation. The HSR’s anti-inflammatory attributes are underpinned by its ability to directly inhibit nuclear factors of the κB family (NF-κB) and interfere with the expression of pro-inflammatory cytokines at the gene regulatory level [6–8]. Notably, HSF1 not only enhances the expression of protein chaperones but also activates over 5200 genes involved in inflammation and energy metabolism, including NF-κB, sirtuin-1 (SIRT1), 5’-adenosine monophosphate-activated protein kinase (AMPK) and peroxisome proliferator-activated receptor-γ coactivator-1α (PGC-1α), just to mention a few [9,10].

However, it is metabolically devastating that, in obesity and type-2 diabetes mellitus, decreased expression of both HSP70 and HSF1 is a common feature detected in adipose tissue, liver, skeletal muscle and vascular beds of patients [1,8,11–18]. Depressed HSR is also evident in menopause-related metabolic dysfunctions [19,20] and in tissues of older adults [21,22], including those presenting neurodegenerative diseases [23]. Impaired HSR has been described in rodent models of obesity, insulin resistance (IR) and cardiovascular disease as well [24–28].

**Figure 1:**
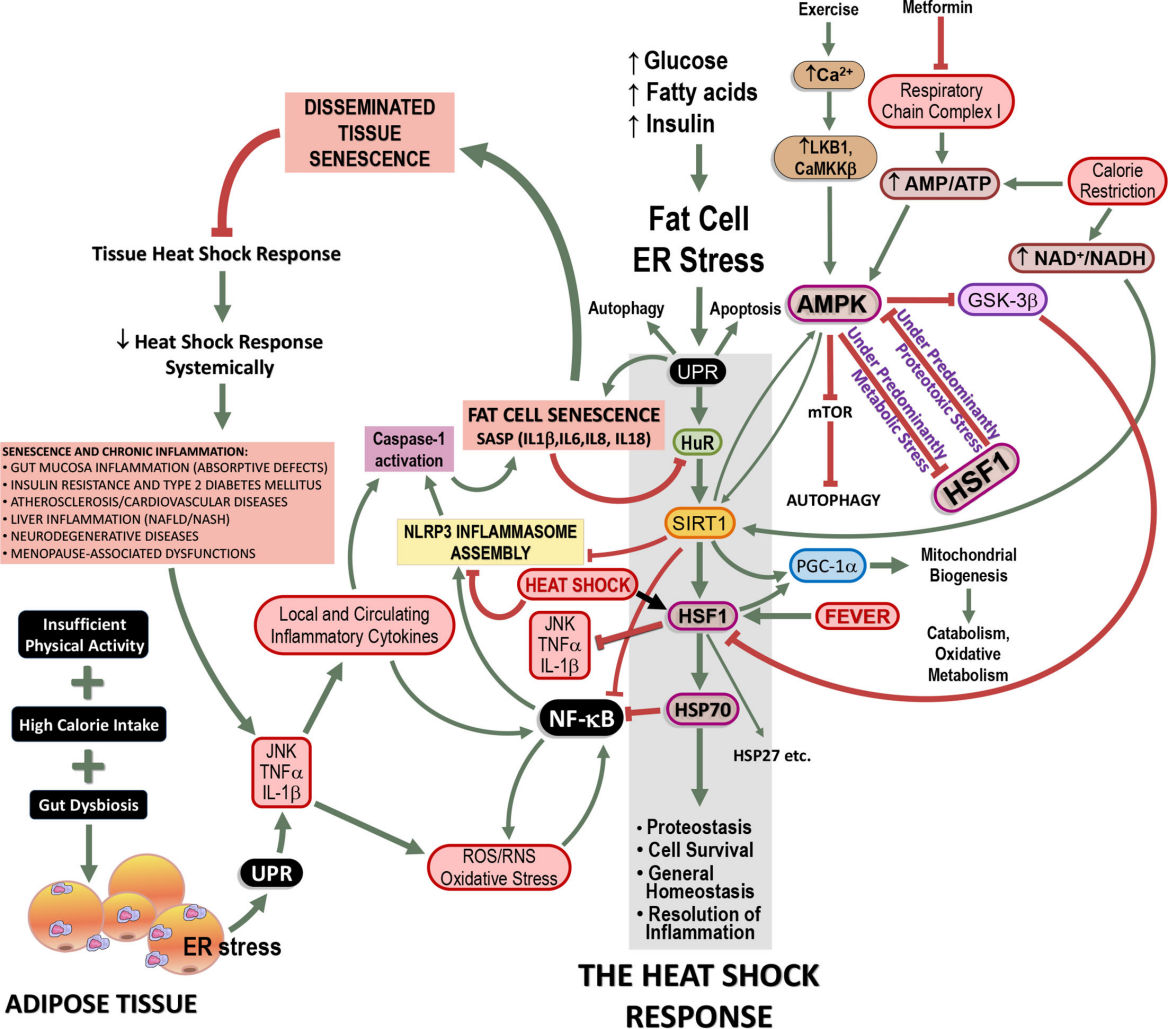
The heat shock response (HSR) and its failure in chronic inflammatory diseases: role of cellular senescence. Under normal nutrient supply (i.e., equivalent to energy expenditure and physical activity), glucose and fatty acids are utilised in the adipose tissue upon physiological amounts of insulin. Any excess of demand is counteracted by enhanced HSR (shaded grey rectangle) in order to supply the proper furnishing of chaperones thus avoiding or correcting endoplasmic reticulum (ER) stress. If ER stress does not cease, it evolves to the unfolded protein response (UPR), which is a cellular strategy primarily aimed at arresting protein synthesis and permitting appropriate protein folding to avoid the formation of potentially toxic protein aggregates. Nevertheless, UPR has an inflammatory branch based on nuclear factors of the κB (NF-κB) family and NLR (especially, NLRP3) inflammasomes. On the other hand, if evolution preserved a cellular response that can lead to a momentary state of inflammation during UPR, cells evolved in parallel an anti-inflammatory strategy based on heat shock factor-1 (HSF1)-dependent expression of the 70 kDa heat shock proteins (HSP70) and other chaperones that convey cells to protein/metabolite homeostasis under predominantly proteotoxic stress, in which HSF1 downstream pathways have prevalence over 5’-adenosine monophosphate-activated protein kinase (AMPK) pathways, which are preferentially activated under predominantly metabolic stress (energy shortage) conditions. This anti-inflammatory pathway is the HSR. However, under a western lifestyle, when adipose tissue becomes overloaded with nutrients and there is no compensatory energy expenditure or skeletal muscle-derived myokines to balance cell metabolism and appetite control, which is aggravated by gut dysbiosis, high amounts of surplus energetic metabolites should be stored in adipose tissue under a higher insulin command leading to uninterrupted ER stress, which is followed by UPR. Under the persistence of risk factors (e.g., excess fatty acids, cholesterol, high-fat diet [HFD] and hyperglycaemia), UPR is diverted to its inflammatory arm in strong positive feedback because continuous inflammatory stimuli do not cease to activate NLRP3 inflammasome, leading to the activation of caspase-1 (pale purple rectangle). Activated caspase-1 determines, in adipocytes, a state of frank cellular senescence which culminates in senescence-associated secretory phenotype (SASP). Proliferative senescence is a cellular alternative of UPR to both autophagy and apoptosis. Nonetheless, SASP spreads out to other tissues and cell types, including adipose tissue infiltrating macrophages, skeletal muscle cells, pancreatic β cells, hepatocytes, vascular cells, and brain structures. In all the above cell types, including adipocytes, SASP leads to the cleavage of HuR, an mRNA-binding protein responsible for enhancing sirtuin-1 (SIRT1) expression. As a consequence, HSF1 expression and transcribing activity become depressed because SIRT1 enhances both. Therefore, HSR is hindered accordingly, and a state of enhanced inflammation is noted because HSR is of crucial importance for the resolution of inflammation. As a healthy HSR cannot resume, the resolution of inflammation is dramatically jeopardised, thus impeding an efficient solution of UPR via HSR. This eventually culminates in the perpetuation of inflammation, which becomes chronic. Besides of this, senescent cells are resistant to undergoing apoptosis (which should be an alternative to breaking this vicious cycle), so chronically inflamed cells are likely to persist in tissues. Such a situation is commonly referred to as low-grade inflammation. As depicted in this illustration, HSR can be physiologically restored by exercise, which is the most powerful HSF1 activator and HSR inducer, comparable only to fever and heat thermotherapy (e.g., heat shock, sauna and hot tubbing). Of note, heat treatment, even in the fever-like range is capable of dismantling NLRP3 inflammasome assembly. Other manoeuvres able to enhance AMPK and SIRT1 activities can help rescue the HSR as well. As the deterioration of the HSR over time is notably linked to the decrease of HSP70 expressions in response to thermal challenges, we systematically examined whole-blood heat challenges throughout HFD treatment in mice, underscoring the impact of HFD on HSR derangement. Reused from: HSR during the resolution of inflammation and its progressive suppression in chronic-degenerative inflammatory diseases. Cell Stress & Chaperones. 2024; 29(1):116–142. https://doi.org/10.1016/j.cstres.2024.01.002 (under the CC BY-NC-ND licence), which is adapted and reused from: Physiological regulation of the HSR by glutamine: implications for chronic low-grade inflammatory diseases in age-related conditions. Nutrire. 2016; 41:17. https://doi.org/10.1186/s41110-016-0021-y under an open access creative common CC BY license 4.0. JNK, c-Jun N-terminal kinases; PCG-1α, peroxisome proliferator-activated receptor-γ coactivator-1α.

The primary cause of HSR failure in both human and animal models is closely tied to the Western-type lifestyle, characterised by high-calorie intake and insufficient physical activity, leading to persistent ER stress. This initial stress in adipose tissue escalates to an unfolded protein response (UPR), triggering an inflammatory cascade through NLR-type inflammasomes, primarily NLRP3, culminating in systemic inflammation. This state promotes proliferative senescence and the senescence-associated secretory phenotype that suppresses the HSR, thereby perpetually blocking the physiological resolution of inflammation via the HSR, as illustrated in Figure 1.

Notably, extracellular HSP70 (eHSP70) production and release are crucial for the anti-inflammatory HSR [21,29]. However, chronically elevated eHSP70 levels correlate with IR, TNFα concentrations, cortisol levels and plasma leptin/adiponectin ratios [30]. High levels of eHSP72 in the plasma correlate positively with IR and can cause pancreatic β-cell dysfunction and death by binding to Toll-like receptors types 2 and 4 (TLR2/TLR4) [30]. Maintaining the balance between eHSP70 and intracellular HSP70 is now accepted to be directly linked to the immunoinflammatory equilibrium of individuals [8,31–34].

Furthermore, the expression of HSR components in peripheral blood leucocytes (PBL), particularly peripheral blood mononuclear cells (PBMC), inversely correlates with pro-inflammatory states in chronic disease in both animals and humans [2,21,22,35–38]. Therefore, to assess the HSR’s status in rodents and humans, we have developed a simple technique involving short-term heat challenges of whole-blood preparations under various conditions. This method provides a less invasive means of evaluating the HSR and aligns closely with tissue HSP70 levels [38], and hence, with organismal anti-inflammatory status.

One crucial consideration is the ambient temperature (T_a_) in which mice are housed, as it significantly affects their basal metabolic rate (BMR), body weight gain and overall metabolism. We house the animals in rooms where the cage bedding level temperature is maintained at 28.5 °C ± 0.5 °C, within the lower critical temperature (LCT) range of the mouse thermoneutral zone (TNZ) [39,40]. Thermoneutrality represents the range of T_a_ where neither heat-conservation nor heat-dissipation mechanisms are activated [20]. For mice, the TNZ spans 30 °C–34 °C, analogous to 21 °C for a lightly clothed human [39]. Within the TNZ, temperature regulation relies solely on sensible heat loss, without metabolic heat production or evaporative cooling adjustments [39]. This raises the question of whether studies with mice below thermoneutrality have any utility in comparison with humans [39–42]. Since many studies house mice at temperatures below their TNZ, which impacts food intake and metabolism [39,40,43], we have begun exploring whether a high-fat diet (HFD) induces significant metabolic changes in our models when mice are housed within the TNZ.

In the present paper, we investigated the progression of bodily HSR capacity in HFD mice housed at a temperature within the TNZ by assessing the magnitude of HSP70 expressions [ΔHSP70 = (HSP70 at 42 °C) **–** (HSP70 at 37 °C)] in whole-blood preparations subjected to heat challenge. We found that, during the 22-week observational period, the ΔHSP70 expression fitted a five-parameter logistic (5PL) regression with the progression of IR predicting the onset of type-2 diabetes in these animals.

## Materials and methods

### Materials

All the chemicals were purchased from Sigma-Aldrich (São Paulo, Brazil) unless otherwise stated. PBS (Sigma) consisted of 136.8 mM NaCl, 2.7 mM KCl, 0.9 mM KH_2_PO_4_, 6.4 mM Na_2_HPO_4_ and pH 7.4.

### Ethics

The investigation followed all ethical rules established by Arouca’s Act (Brazilian Federal Law no. 11794/2008) and the 8th Edition of Guide for Care and Use of Experimental Animals published by National Research Council of the National Academies (2011; available at https://grants.nih.gov/grants/olaw/guide-for-the-care-and-use-of-laboratory-animals.pdf) in accordance with the ARRIVE guidelines and the NIH’s Principles and Guidelines for Reporting Preclinical esearch (Available from www.nih.gov/research-training/rigor-reproducibility/principles-guidelines-reporting-preclinical-research, accessed 16 November 2023). All the procedures (protocols #23,451 and #37,881) were reviewed and approved by the Committee of Animal Welfare of the Federal University of Rio Grande do Sul, which adheres to the Guidelines of the Brazilian National Council for the Control of Animal Experimentation.

### Animals

Adult (90 days old) C57BL/6 J mice (males and females) purchased from The Jackson Laboratories (Bar Harbor, ME, U.S.A.) and inbred at The Federal University of Rio Grande do Sul Institute of Basic Health Sciences Animal Care Facility (CREAL) were randomly assigned (https://www.graphpad.com/quickcalcs/randomN2/) and used. The animals were maintained under controlled temperature (25 °C ± 2°C) and humidity (60 %) in a 12/12-hour light/dark cycle (lights on at 07:00 hours), having free access to water and pelleted rodent standard chow (NUVILAB CR-1, Quimtia, Colombo, Brazil).

**Figure 2:**
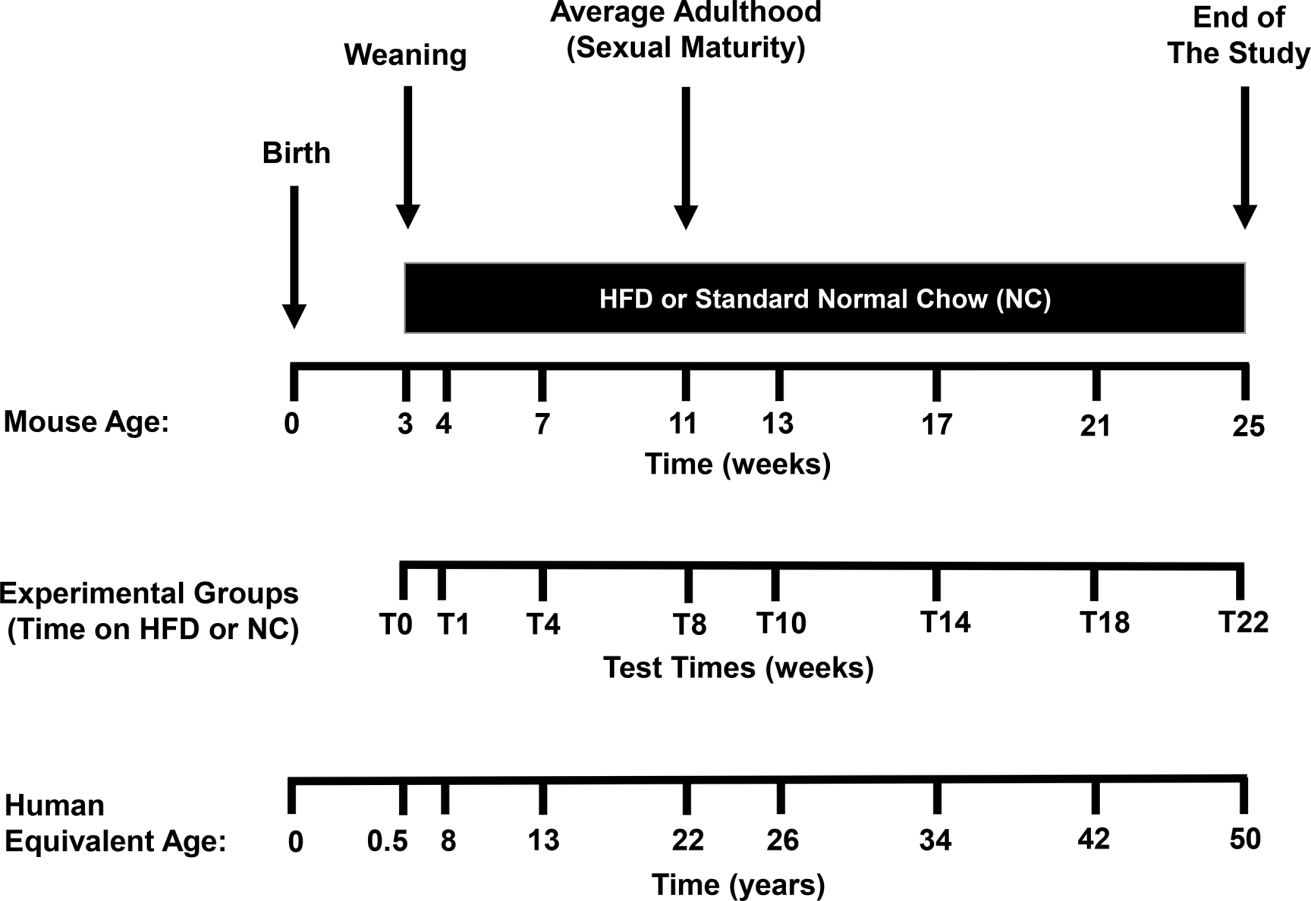
Study design for diet interventions. Just-weaned (21 days old) male C57BL/6 J mice were randomly assigned to be fed a high-fat diet (HFD, *n*=48) or normal standard chow (NC, *n*=48). Animals were followed for up to 22 weeks and killed at the time points indicated (from T0 to T22 weeks; *n*=12 per time point, i.e., 6 controls and 6 on HFD). The age equivalence in humans [41] is given at the bottom of the figure.

For diet intervention studies, just-weaned (21 days old) male C57BL/6 J mice were randomly enrolled by simple randomisation (https://www.graphpad.com/quickcalcs/randomN2/) to be fed *ad libitum* on either NUVILAB CR-1 standard normal rodent diet (total metabolisable energy: 16.6 MJ/kg, being 11.4 % as fats, 62.8 % as carbohydrates and 25.8 % as proteins), hereafter referred to as normal chow (NC), or lard-based HFD consisting of 22.8 MJ/kg total metabolisable energy (58.30 % as fats, 24.25 % as carbohydrates and 16.45 % as proteins), adapted from reference [44]. All diet ingredients (e.g., fibres and micronutrients) in the HFD (except for starch and lard) were adjusted to be present in the same amount as in the standard chow [45]. Mice were maintained under controlled temperature (25 °C ± 2°C) and humidity (60 %) in a 12/12-hour light/dark cycle (lights on at 07:00 hours), being housed (5 mice per cage) in autoclavable polypropylene plastic cages (40 × 33 × 17 cm; 1,110 cm^2^ internal area), having free access to food and water during the experiments, unless otherwise stated. Notably, the temperature measured at the cage bedding level was 28.5 °C ± 0.5 °C, within the LCT range of the mouse TNZ [39]. This allows for a more realistic extrapolation of the results to humans [39–41,43]. The animals were followed for up to 22 weeks and killed at the time points outlined in Figure 2, ranging from T0 to T22 weeks. The equivalent human years [46] are given at the bottom of the figure. The experimental timeframe was selected to emulate the adult phase of humans living a western lifestyle.

**Figure 3:**
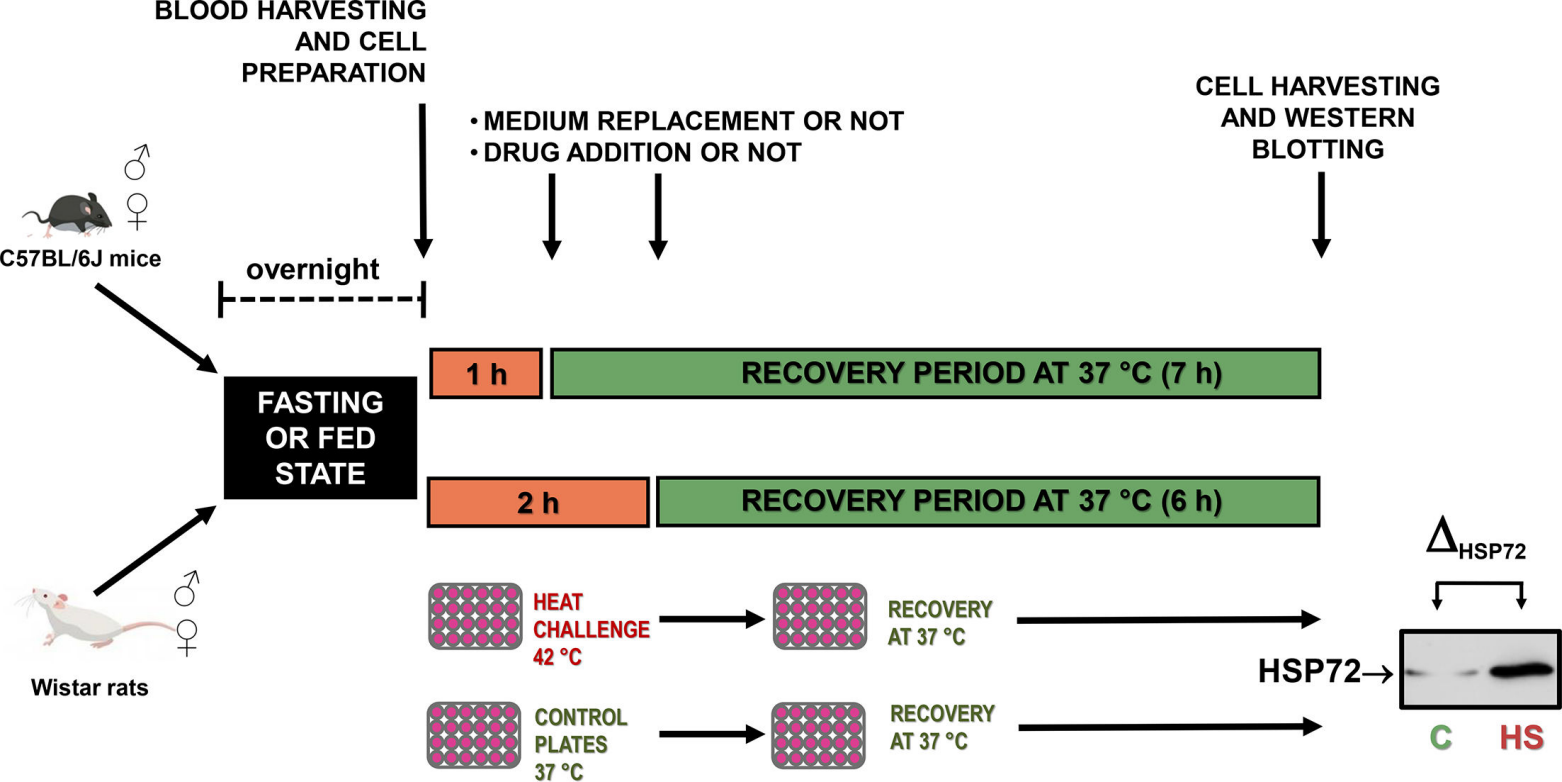
Heat challenge experimental design. To optimise the conditions for the *ex vivo* whole-blood challenge test, we collected samples from both male and female mice and rats, either after an overnight fast or without fasting. These whole-blood samples were then mixed with a culture medium and exposed to heat challenges at 42 °C, while control samples were maintained at 37 °C. Heat challenges lasted for either 1 or 2 hours. Subsequently, the cells were given 6 or 7 hours to recover (total incubation time of 8 hours) at 37 °C, allowing for the accumulation of the inducible forms of HSP70 (HSP72), a key indicator of heat shock response (HSR) capacity. After haemolysis, leucocytes were later subjected to electrophoresis for HSP70 detection through immunoblot analysis. Throughout this experimental protocol, we also explored the effects of culture medium replacement and the addition of specific drugs. These variables included the washing of cells and the introduction of fresh medium, or the retention of the original medium, either immediately after blood collection or following the heat (or control) challenge, as indicated by the arrows in the experimental timeline. Comprehensive information about the quantity, age, and sex of the animals utilised can be found in the figure legends. **Adapted from:** Resolution of Inflammation in Chronic Disease via Restoration of the HSR. Cell Stress and Chaperones. 2024; 29(1):66–87. https://doi.org/10.1016/j.cstres.2024.01.005 (under an open access CC BY-NC-ND licence).

For the preliminary tests designed to set up the best conditions for whole-blood heat challenge (Figure 3), adult (90 days old) C57BL/6J mice (males and females) were employed. In addition, adult (60 days old) male Wistar rats obtained from CREAL, maintained under controlled temperature (23 °C ± 1 °C) and humidity (60 %) in a 12/12-hour light/dark cycle (lights on at 07:00 hours) and housed in plastic cages (49 × 34 × 16 cm), were also used during this phase of the studies. Throughout the experiments, the animals had free access to water and were fed standard NUVILAB CR-1 (Curitiba, PR, Brazil) chow *ad libitum*. We opted to utilise rats in the initial standardisation tests as the quantity of blood obtained from a single animal is sufficient for conducting experiments that require the analysis of multiple samples from the same subject, such as those investigating the effects of insulin, phenylephrine and prazosin in whole-blood heat challenges. The protocol using rats instead of mice for some preliminary studies was suggested by the Ethics Committee in order to adhere to the 3R Principle for conducting animal research more humanely since our previous studies had shown that rats respond equally to our manoeuvres as mice do.

The number of animals in each experiment is provided in the respective figure legends. There was no exclusion of any animals, data or samples during all the experiments. Moreover, all the outcomes are reported in the Results section. In addition, all the following analyses were performed blindly by investigators unaware of the groups.

The animals, evaluated by the attending veterinarian, were in excellent health and showed no signs of infection during the studies. They were also test-naïve.

### Whole-blood heat challenge protocol

Before the experiments, the animals were either fasted overnight or fed *ad libitum* to be killed the next morning. Blood was collected in heparinised tubes and immediately diluted (1:10 by volume) in RPMI1640 culture medium (Sigma, code R6504, pH 7.4, containing 2 mM l-glutamine, 24 mM NaHCO_3_, 11 mM d-glucose, 100 U/mL penicillin and 100 μg/mL streptomycin) to be pipetted into 24-well plates (~2 mL final volume). Then, the plates were submersed in a temperature-controlled water bath (Precision Scientific, U.S.A.) at 42.00 °C ± 0.01 °C for either 1 or 2 hours (immersion depth = 2.0 cm; *t*_1/2_ = 1.5 min) as previously described [2]. Parallel control preparations were maintained in another water bath at 37 °C for the same time periods. After water bath incubations, cells from both control and heat-treated groups were incubated for an additional 6 or 7 hours (depending on the above heat treatment period) at 37 °C in a 5 % (v/v) CO_2_ humidified atmosphere in air to allow for a robust heat-induced accumulation of HSP70 (actually, HSP72 = *hspa1a* and *hspa1b* gene products). The extent of the HSR, i.e., the degree of HSP70 upregulation in whole-blood preparations subjected to heat challenge, was quantified by the following equation: ΔHSP70 = (HSP70 at 42 °C) – (HSP70 at 37 °C). Therefore, ΔHSP70 serves as a marker of the HSR capacity [37]. Additionally, in calculating ΔHSP70, the HSP70 expressions at 42 °C and 37 °C (both relative to GAPDH expressions) were normalised using the highest value observed among all animals in each experimental round, resulting in the maximum value being unity for each round. The total experimental time, from blood harvesting to the end of incubations, was 8 hours.

The utilisation of a cell culture system employing whole blood circumvents a prior extraction of erythrocytes and the subsequent loss of mononuclear cells during the purification process using isopycnic gradients [21,36]. Additionally, this approach averts the depletion of growth factors and other co-factors inherent in animals’ blood, enabling cultivation with autologous factors. Consequently, there is no necessity to supplement cultures with crucial elements such as growth factors, serum, hormones and cytokines essential for the immune response [47].

During this protocol, we also tested the effects of culture medium replacement prior to the final 6- or 7-hour incubations, by washing cells and adding fresh medium either just after blood collection or after the heat (or control) challenge, as illustrated in Figure 3. Cell viability, assessed after heat (or control) challenge and at the end of the experiment, was always higher than 95 %, as inferred by the Trypan blue dye exclusion.

The rationale for choosing the present heat shock challenge model, operating within the fever-like physiological temperature range *in vivo*, derives from findings in our laboratory and others. Heat treatment of whole blood in vitro for 2 hours at varying temperatures (39 °C, 41 °C and 42 °C) demonstrates that HSP70 expression depends on both temperature and cell type. Monocytes showed significant induction at 39 °C, with HSP70 levels at 41 °C being 10 times higher than at 37 °C. In contrast, lymphocytes and polymorphonuclear leucocytes exhibited only modest increases up to 41 °C. A 3-hour recovery period was used to assess HSP70 in PBL [48]. Lymphocytes, which account for nearly all PBL HSP70 expression [49], displayed no induction at 39 °C, a slight increase at 41 °C and a marked 500 % increase at 42 °C compared with controls at 37 °C [48]. Unlike monocytes and PBMC, lymphocytes typically require temperatures above 42 °C for significant HSP70 expression. This may reflect the limited activation of HSF1 and DNA binding below 41 °C in lymphocytes. At higher temperatures, HSF1 activity is linked to NF-κB inhibition, peaking between 43 °C and 45 °C [50]. In naïve cells (not previously heat-shocked), HSP70 mRNA transcription after a strong heat shock (43 °C for 1.5 hours) peaks between the end of the heat shock and 1 hour of recovery at 37 °C, with maximal HSP70 protein accumulation occurring at 4–6 hours [51].

*Ex vivo* heat treatment of whole blood from healthy human volunteers involved incubation at 37 °C, 38 °C, 39 °C, 40 °C and 41 °C for 90 min, followed by recovery at 37 °C. HSP70 was measured in PBLs at 0, 2, 4, and 6 hours post-treatment. At control (37 °C) and lower temperatures (38 °C–39 °C), HSP70 levels rose to 4 hours post-heat shock and returned to baseline by 6 hours [52]. A typical HSR, characterised by HSP70 accumulation after a 2-hour heat challenge, peaks at 42 °C, with smaller increases at 37 °C–39 °C and declines below baseline at 45 °C [49].

When cells were heat-shocked at 42 °C for up to 5 hours, HSP70 levels peaked at 6 hours post-treatment. This heat challenge maximally inhibited viral proliferation, including HIV-1, in an HSP70-dependent manner, similar to the effects of HSP70-inducing compounds like cyclopentenone prostaglandins (cyPGs) [53,54]. Even at sublethal temperatures (45 °C for 10 min), maximal HSP70 accumulation occurred after 6 hours of recovery at 37°C, coinciding with cyPG effects on HSP70 [55].

For our *ex vivo* heat challenge experiments, a 2-hour heat shock was selected, as HSP70 release from PBMC peaks during this time [49], consistent with observations in Jurkat T cells [56]. Under these conditions, HSP70 expression typically peaks between 2 and 4 hours and returns to baseline by 6 hours post-treatment [52]. This protocol produces robust iHSP70 accumulation and HSP70 release. In our experiments, whole-blood preparations incubated at 42 °C for 2 hours exhibited a significant 50 %–75 % increase in **hspa1a** and **hspa1b** gene products of HSP70, but no significant increase was observed after 1 hour.

### Evolution of body weight, obesity, energy intake and metabolic efficiency

For diet intervention studies, mice were randomly allocated to either NC or HFD groups and were killed at the time points illustrated in Figure 2, spanning from T0 to T22 weeks of diet intervention. All animal-related procedures, including euthanasia and measurements, were conducted by an observer unaware of the experimental group assignments.

Food intake and body weight were monitored weekly, and the nasoanal length of each animal was measured to calculate the ‘nutritive’ (or Lee) index [57–59], which is an approximate measure of rodent obesity [27]. The Lee index is derived from the formula (body weight)^1/3^ × (nasoanal length)^−1^, with weight in grammes and length in centimetres. These measurements were also assessed weekly.

### Progress of glycaemic status and IR

The progress of glucose intolerance and the onset of IR in HFD mice as compared with NC animals were evaluated through an oral glucose tolerance test (oGTT; [60]) and intraperitoneal insulin tolerance test (ipITT; [45,61]), 1 week before the death of animals in each group, except for the T0 group, when the procedure was performed at weaning. For oGTT (always in the afternoon, approx. 13:00 hours), the mice were fasted for 6 hours prior to gastric gavage of about 400 μL (10 μL/g of body weight) of an 80 % (w/v) glucose solution in PBS, equivalent to 1.25 mL/kg of body weight (1 g/kg body weight final glucose dose), and then 0.5 μL blood samples were collected from the tail at time zero (baseline), 15, 30, 60, 90 and 120 min after the glucose load. For ipITT (also in the afternoon but with 72 hours of difference from the days of oGTTs), the animals were fasted for 6 hours and received an intraperitoneal injection of approximately 150 μL (5 μL/g body weight) of a 100 IU/mL insulin (Humalog Lispro – Lilly) solution in PBS, equivalent to 1 IU/kg, and then 0.5 μL-blood samples for glycaemic measurements were collected from the tail at time zero (baseline), 15, 30 and 45 min after insulin administration. To obtain blood for glycaemic tests, the animals’ tails were neither cut nor punctured with a lancing device. Instead, a small piece of hair from the tip of the tail was gently plucked, yielding a drop of blood for use in the tests, employing Optium Xceed Glucometer (Abbott).

To assess the extent of glycaemic alterations induced by diet manipulation, incremental areas under glycaemic curves (iAUC) in oGTT were individually measured for each animal, and the area under the glycaemic curves was subtracted from the area under the baseline through the trapezoid method [62,63], where any area beneath the fasting level is ignored. In this latter case, for the portion of the time interval when blood glucose is above the fasting level, the actual area was calculated geometrically using the trapezoid method by the similarity of triangles (from Thales’ theorem [64]). The mean ± S.D. of the individual iAUC of all the animals for each time of blood collection was used to build up the glycaemic curves of the groups. The same applies to the inverted incremental areas under the curves for ipITT (inv-iAUC), where any value above the baseline was discarded, and any intermediate portion of the curve was resolved by Thales’ theorem [63]. In addition, we were also aware that during this short insulin tolerance test, the fall in plasma glucose after insulin injection is not only due to an increased rate of glucose uptake in tissues but also to a reduced rate of output of glucose from the liver [65].

Apart from oGTT and ipITT curves and their respective AUC, fasting glycaemia, fasting insulinaemia and 2 hours post-glucose load glycaemia in oGTT were also considered for comparisons.

### Estimation of glycaemic status and the appearance of IR

Although HOMA-IR has been commonly utilised as an estimate of glycaemic status in humans, numerous studies have been conducted to validate its applicability in both rats and mice. Notably, investigations into insulin sensitivity during pregnancy in Wistar and Sprague-Dawley rats [66], cardiovascular risk biomarkers and diabetic cardiomyopathy in insulin-resistant streptozotocin-type-2 diabetes mellitus rats [67], β-cell dysfunction in female Sprague-Dawley rats [68] and a dedicated study validating HOMA-IR in an HFD-induced insulin-resistant model in Wistar rats [69] have revealed that HOMA-IR is a valid measure to determine IR in Wistar rats exhibiting similarities between human and rat models.

Although our present work is not dedicated to proposing any translational association between surrogate indices of glycaemic status between mice and humans, similar validations have been undertaken in mice, notably in the work by Winzell and Ahrén characterising impaired glucose tolerance (IGT) and type-2 diabetes mellitus in C57BL/6J mice fed an HFD [44], which is the model we have been using. Comparative analyses between surrogate indexes of insulin sensitivity and resistance, as well as hyperinsulinaemic euglycaemic clamp estimates in C57BL/6J and FVB mice, have demonstrated comparable behaviours with human HOMA-IR and QUICK indices [70]. A comprehensive study involving over 100 mouse strains, including C57BL/6J on a HFD and/or high-sucrose diet, investigated HOMA-IR and performed euglycaemic-hyperinsulinaemic clamp studies on the prototypical laboratory mouse strain, C57BL/6J, revealing similarities to human responses [71]. Insightfully, studies, such as those by Shu and colleagues, have explored HOMA-IR and QUICKI values in HFD-induced IR in mice [72].

In our current study, we incorporated both male and female animals, recognising significant differences in IR between the sexes. Notably, in C57BL/6 J mice, the HOMA-IR index in males can reach a staggering 50, while in females on an HFD, the maximum is 5 [71]. This sex-specific variability holds pivotal importance for interpreting results extrapolated to humans, given the consistent profile and range of values the HOMA-IR index demonstrates when comparing rodents and humans [69]. However, it is essential to clarify that our aim was not to extrapolate mouse glycaemic status results to humans. On the contrary, our focus was on comparing the HSR and glycaemic status during the period of HFD treatment. Additionally, it is worth mentioning that we opted to use male mice in glycaemic studies based on our preliminary experiments (see Results), revealing an equally intense HSR in both male and female mice.

In addition to the above explanations, Fraulob and co-workers [73] have employed a mouse model of metabolic syndrome, IR and non-alcoholic fatty liver disease in C57BL/6 J mice fed an HFD, incorporating HOMA-IR for mice. Consequently, in our study, we utilised HOMA-IR and QUICK indices alongside fasting glycaemia, fasting insulinaemia and 2-hour postload glycaemia. HOMA-IR values were calculated using the equation by Matthews and colleagues [74]: fasting plasma insulin (μU/mL) × fasting plasma glucose (mmol/L)/22.5. The QUICK index (QUICKI) [75] was determined by the equation: QUICKI = 1/[log(I_0_) + log(G_0_)], with I_0_ representing fasting insulin (μU/mL) and G_0_ being fasting glycaemia (mg/dL). A HOMA-IR score above 2.5 was assumed to be indicative of IR, akin to humans, while QUICKI values between 0.450 and 0.339 denote normal glycaemic status, and values below 0.339 suggest IR.

Interestingly, although HOMA demonstrated a high correlation with QUICKI, the overall correlation between the gold standard SI_Clamp_ and QUICKI surpassed that between SI_Clamp_ and HOMA [75]. Both HOMA-IR and QUICK indices, derived independently using empirical methods, exhibit a robust mathematical relationship. The strength of these measures as surrogates depends on the underlying physiological relationship between hepatic insulin responses (i.e., suppression of hepatic glucose production by insulin under fasting conditions) and whole-body insulin responses measured with the clamp procedure (largely reflecting skeletal muscle insulin responsiveness [76]). This latter editorial referenced the work of Cacho and colleagues [66] with rats and of Lee and co-workers [70] with mice.

### Plasma measurements

Insulin was assessed by using a mouse/rat-specific competition ELISA kit (Bertin Pharma, France #A05105 formerly SPI-Bio/Cayman Chemical #589501) in heparinised (12.5 USP units/mL final concentration) mouse plasma samples. ELISA detection limit is 0.08 ng/mL, and the assay range is 0.08–10.00 ng/mL; interassay CV: 6.7 %–8.6 %; intra-assay CV: 0.8 %–2.3 %. In order to allow for comparisons, original nanogram per millilitre values were converted into pmol/L of mouse insulin (5.8 pg/fmol) and then into human equivalent units, considering human insulin molecular weight as 5807.57 g/mol and the conversion ratio of 5.975 fmol/μU of human insulin [77], so that 1 μU/mL = 5.975 pM.

### Euthanasia

The animals (12-hour fast, unless otherwise stated) were killed by decapitation. Euthanasia always occurred in a separate laboratory environment with exhaustion to avoid the influence of fear pheromones [25,26] and was conducted by an experienced researcher. Between the death of an animal and another, the guillotine and the rest of the material were completely sanitised with water, detergent and alcohol. Considering the need to obtain peripheral blood for the analysis of HSR components, animal killing under anaesthesia, while desirable, is incompatible with the experimental goals because all anaesthetics commonly employed with experimental animals lead to intense and sustained hyperglycaemia in rodents [78–80]. Moreover, these anaesthetics interfere with the function of cells involved in the production of HSP72, such as leukocytes [81]. Considering also that plasma concentrations of HSP72, which is a major molecular object of the study, are greatly suppressed by high levels of plasma glucose [82], animal killing was performed without anaesthesia.

### Protein separation and immunoblotting

Following whole-blood challenge incubations, cells were transferred from the 24-well plates to 1.5 mL microfuge tubes, pelleted (370 × *g* for 10 min) at room temperature (22 °C) and washed in ice-cold PBS (370 × *g* for 10 min at 4 °C). Subsequently, pellets were manually disrupted, and erythrocytes were lysed by resuspending cell pellets in 300 μL of Tris-NH_4_Cl (17 mM Tris, 144 mM NH_4_Cl and pH 7.3) for 5 min on ice [83]. The resulting suspension was diluted to 1.5 mL with ice-cold PBS for washing the NH_4_Cl-induced haemolysate and then centrifuged (370 × *g* for 10 min at 4 °C) on an ice bath [84]. After washing, cells were resuspended in PBS and disrupted using a model UIS250V ultrasonic processor (Hielscher Ultrasonics GmbH, Germany) equipped with an LS24d3 sonotrode operating at 24 kHz and 75% of maximal potency (9 W final power in the tubes), in pulses of 0.5 s each for 30 s. The lysis buffer (similar to RIPA buffer) comprised 0.1 % (w/v) SDS containing a freshly prepared protease and phosphatase inhibitor cocktail (Sigma) with final concentrations of leupeptin (4.2 μM), aprotinin (0.31 μM), TLCK (*N*-Tosyl-l-Lysine Chloromethyl Ketone, hydrochloride; 20 μM), PMSF (Phenyl-Methyl-Sulfonyl Fluoride, 100 μM), sodium orthovanadate (Na_3_VO_4_; 1 mM), sodium molybdate (Na_2_MoO_4_; 1 mM) and β-glycerophosphate (1 mM). Ten microlitres from cell lysates were reserved for protein determination [85], and the remaining samples were stored at −86 °C until use. Upon thawing, they were processed for SDS-PAGE, as described in [14], with modifications [2].

For protein separations, equivalent amounts (~40 μg) of protein from whole-cell lysates were mixed with 2× Laemmli’s loading buffer [50 mM Tris, 10 % (w/v) SDS, 10 % (v/v) glycerol, 10 % (v/v) 2-mercaptoethanol and 2 mg/mL bromophenol blue, final concentrations] in a 1:1 ratio, boiled for 5 min and electrophoresed (Mini-Protean Tetra Vertical Electrophoresis Cell, Bio-Rad, 1000 Alfred Nobel Drive Hercules, California 94,547 U.S.A.) in a 10% polyacrylamide minigel for 4 hours at 15 mA/gel. The proteins were then transferred onto nitrocellulose membranes (GE HealthCare) according to the electrotransfer manufacturer’s (Bio-Rad) instructions (2 hours and 100 V), and transferred bands were visualised with 0.3 % (w/v) Red Ponceau S (Sigma) in a 3 % (w/v) trichloroacetic acid solution to be photodocumented (ImageQuant 350, GE HealthCare).

For immunoblotting procedures, the SNAP i.d. (Merck Millipore) quick immunoblot vacuum system was utilised [16]. The membranes were washed with water, blocked in 1 % (w/v) BSA (fraction V, Sigma, A2153) in wash buffer [50 mM Tris, 5 mM EDTA, 150 mM NaCl (TEN) Tween 20 (0.1 % w/v) solution and pH 7.4] for 30 s, and then washed three times (30 s each) with washing buffer. Subsequently, the membranes were incubated for 10 min with mouse anti-human HSP70 monoclonal antibody (Sigma H5147, clone BRM 22 ascites fluid), recognising both the 73 kDa ‘constitutive/cognate’ HSC70 (*hspa8* gene product) and the 72 kDa ‘inducible’ (*hspa1a* and *hspa1b* gene products) forms of the HSP70 of human, mouse and rat origin, at a 1:5,000 dilution (by volume) in washing buffer. After three washes with washing buffer (30 s each), membranes were probed with biotin-labelled goat anti-mouse IgG whole molecule (Sigma B7264, at 1:80,000 dilution) and then incubated with horseradish peroxidase-labelled ultrasensitive streptavidin polymer (Sigma, S2438, at a 1:1,000 dilution). Protein detection was performed using the enhanced chemiluminescence method (ECL Prime, GE HealthCare). Immunocontents were normalised in terms of GAPDH expression (rabbit anti-mouse GAPDH IgG, Sigma G9545, at a 1:1,000 dilution, followed by goat anti-rabbit biotinylated IgG, Sigma B8895; at a 1:1,000 dilution). Protein bands were photodocumented for 600 s (60 frames, 1 photo every 10 s) and quantified in ImageQuant 350 chemiluminescence system (GE HealthCare) and the accompanying online stacking imaging software ImageQuant TL 7.0. The data are presented as the means ± S.D. of immunocontents in terms of GAPDH gel loading controls. Prestained molecular standard protein ladders (cat. no. 1,610,395 Precision Plus Protein Kaleidoscope, Bio-Rad) were employed. All the antibodies used were previously validated in studies conducted by our group [2,16].

The densitometric results presented in all figures involving immunoblots were normalised as follows. First, the densitometric values for HSP70 (either HSP72, HSP73 or total HSP70 = HSP72 + HSP73) were divided by the corresponding GAPDH densitometry values for each lane, producing the initial normalisation for that gel. Subsequently, these initial normalised values were divided by the highest value among all lanes within the same gel, giving the secondary relations. As a result, the maximum normalised value becomes unity, with all other values expressed as fractions between 0 and 1. The means ± S.D. of these secondarily normalised values across all runs were calculated and are presented in the figures.

### Sample size

The sample size was calculated to detect the smallest expected difference between control and HFD-treated animals (50 % ± 20 % change for the main critical variable of the study, the relative HSP70/GAPDH expression; [45]). A statistical power of 80 % was used for the calculation for a significance level of *P*<0.05 and the DIMAM 1.0 Sample Dimensioning software for Windows from Editora Guanabara Koogan. The number of animals used for the execution of the study was the minimum necessary to produce conclusive results, saving the animal from suffering as much as possible and strictly adhering to the principle of 3Rs (The Principles of Humane Experimental Technique), making a total of eight mice per group to assess whole-blood HSR (total 40 males and 16 females) during the standardisation phase, and then, six male mice per group were enrolled for HFD experiments in eight-time groups (T0, T1, T4, T8, T10, T14, T18 and T22 weeks; total 48 animals in control NC diet and 48 in HFD). Notably, although mice from all the above eight-time groups have been submitted to all glycaemic status studies, animals from T0 (just-weaned) were excluded from whole-blood heat challenge studies because the amount of blood is insufficient to perform the tests.

For the preliminary heat challenge studies in the presence of insulin, phenylephrine and prazosin, 10 adult male rats were also used for the reasons stated in the Animals section.

### Replication statement

In the initial experiments aimed at optimising conditions for the whole-blood heat challenge, we conducted four replicates, each consisting of four fasting females, four fasting males, four fed females and four fed males, resulting in a total of 64 samples. All four replicates yielded consistent and identical results. For our dietary intervention studies, we maintained eight parallel replicates for each group (NC and HFD) across the various time points (T0, T1, T4, T8, T10, T14, T18 and T22 weeks) while assessing glycaemic status and HSR parameters. The results were identical across each round of tests.

### Statistical analyses

Before statistical analysis, the outcome variables were assessed for normality through the Kolmogorov-Smirnov test. As there is a wide range of data dispersion, generalised models were initially performed. The generalised linear model (GLM) was used for variables without correlated errors, and the generalised estimated equations (GEEs) were utilised for comparison with more than one measure of each animal. Gama and linear models were tested for both GLM and GEE with an unstructured covariance matrix. The model applied was chosen by the lower value of goodness of fit in the Akaike Information Criterion or *quasi-likelihood* under the independence model criterion for GLM and GEE, respectively. For all these pre-analyses, the pairwise test with Bonferroni *post hoc* was performed, and Statistical Package for Social Science Professional software version 25.0 (IBM Corp., Armonk, NY, U.S.A.) was employed; differences were considered significant when *P*<0.05.

Afterwards, for the comparisons with and within groups of heat challenge (males × females; fasting × fed state; medium replacement or not; insulin addition and phenylephrine/prazosin additions), an ordinary two-way analysis of variance (ANOVA) followed by Tukey’s multiple comparisons test was used. For the analysis of body weight progress, energy intake, Lee index and metabolic efficiency, repeated measures ANOVA or mixed model and Sidak’s multiple comparisons test were utilised. For the analysis of the evolution of HSP70 expressions during the test times in both NC and HFD mice, two-way ANOVA and Sidak’s multiple comparisons test were used.

For the glycaemic curves shown in oGTT and ipITT, which are repeated measures (RM) tests [63], RM ANOVA followed by Tukey’s multiple comparisons test was used. Comparisons between NC and HFD groups with respect to test time evolution of fasting glycaemia, fasting insulinaemia, QUICK index, HOMA-IR, iAUC of oGTT, 2-hour post-load glycaemia and inverted incremental area under the curve (inv-iAUC) of ipITT were conducted using two-way ANOVA and Tukey’s multiple comparisons tests. The correlations of the progress of test time and fasting glycaemia, fasting insulinaemia, QUICK index and HOMA-IR between NC and HFD groups were analysed by the least-square regressions and Tukey’s multiple comparisons tests.

Building upon our previous findings [2], which revealed a 5PL regression-type correlation between alterations in HSP70 expressions in blood lymphocytes [i.e., ΔHSP70 = (HSP70 42 °C) **–** (HSP70 at 37 °C)] and the immunoinflammatory status, we embarked on an exploration of whether the glycaemic status and the progression of IR in both NC and HFD mice could also exhibit a 5PL regression with respect to ∆HSP70. To accomplish this, we employed the least-square asymmetric five-parameter best-fitting subroutine in GraphPad Prism 8.0.1 software, ensuring the exclusion of random effects with zero S.D. from the models. We then cross-validated our results using the online curve fitting software, MyCurveFit (https://mycurvefit.com/). The same approach was applied to establish 5PL regressions between ∆HSP70 and key variables such as fasting glycaemia, fasting insulinaemia, QUICK index, HOMA-IR, iAUC in oGTT, 2-hour postload glycaemia and inv-iAUC in ipITT.

For the calculation of *R*^2^-values from 5PL-fitted curves whose parameters generate the smallest weighted sum of squared errors and the distribution of the responses is approximately normal [86], we utilised a method to normalise Maddala’s *R*^2^, yielding the maximum adjusted *R*^2^-analogue of McFadden’s *R*^2^. Thus, *R*^2^ was calculated by normalising Maddala’s *R*^2^ to obtain the maximum adjusted *R*^2^-analogue [87] of McFadden’s *R*^2^ [88] by using residual QQ plots generated in Prism software. Furthermore, the type-I error *P*-values of the 5PL curves were computed through a *χ*^2^ value, determined as two times the logarithm likelihood of the fitted values minus the logarithm likelihood of the overall probability, with 1 degree of freedom to account for the two models: fitted and overall. This meticulous analytical process enabled us to unveil and assess the intricate relationships between these parameters, shedding light on potential correlations and providing a deeper understanding of our data. Details on specific statistical treatments are given in the figure legends, and data are presented as means ± S.D.

## Results

### Characterisation of the whole-blood heat challenge model

**Figure 4:**
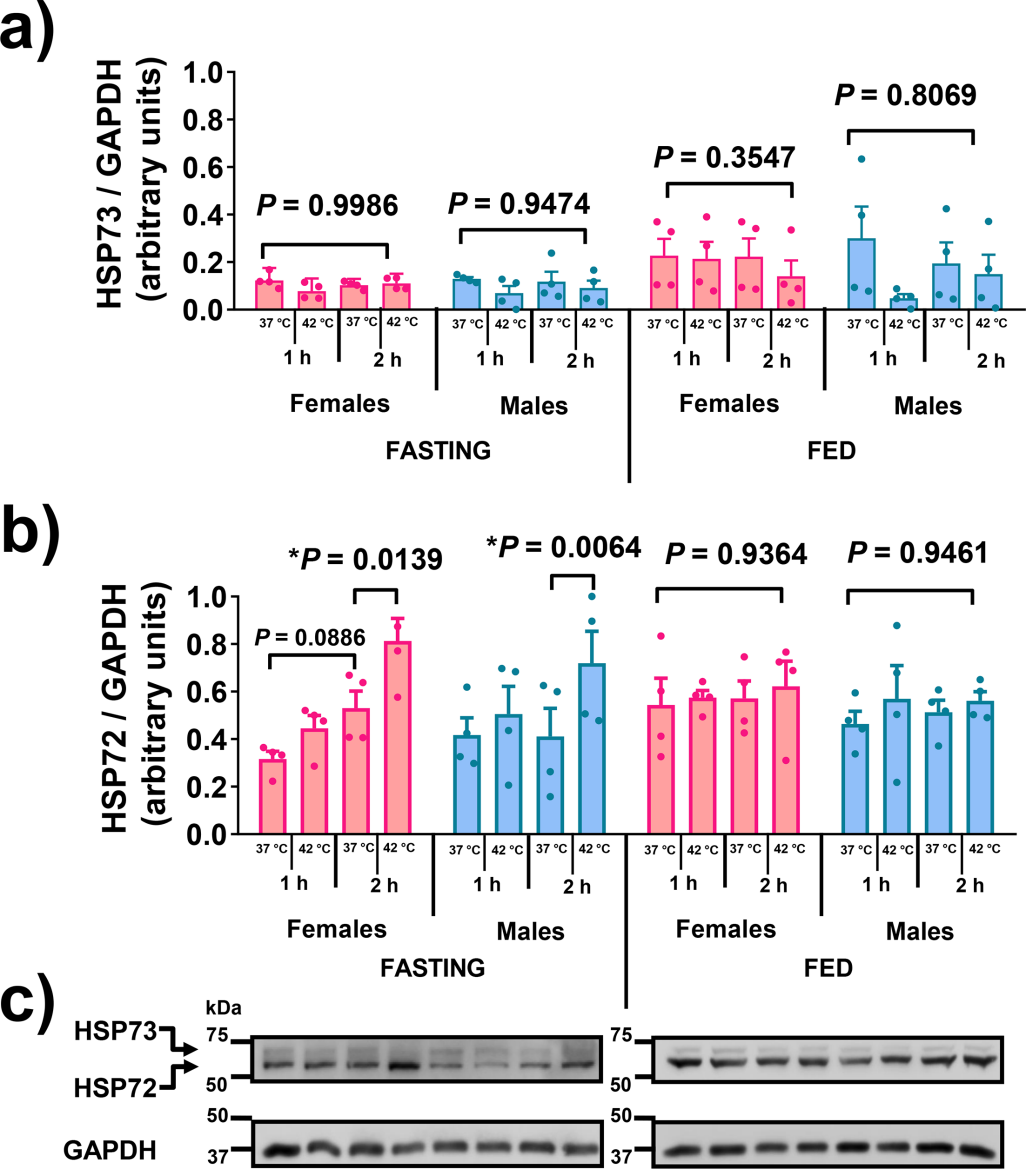
*Ex vivo* whole-blood heat shock challenge. In this experiment, we used 90-day-old adult mice, both male (*n*=32) and female (*n*=32). They were either fasted overnight or kept in the fed state (*ad libitum*). Following decapitation, blood samples were collected in heparinised tubes and immediately diluted 1:10 (by volume) with a culture medium. Subsequently, the samples were pipetted into 24-well plates and subjected to incubation in a temperature-controlled water bath, with options of either 37 °C or 42 °C, and for durations of either 1 or 2 hours. After the heat challenge, the cells were allowed to recover at 37 °C for a total of 8 hours, as explained in the legend of Figure 2. Depicted are the relative expressions of **(a)** the ‘cognate/constitutive’ HSP73 (hspa8) and **(b)** the ‘inducible’ HSP72 (hspa1a and hspa1b) forms of HSP70 in relation to GAPDH. The expressions are presented as means ± S.D. Representative gels **(c)** demonstrate the separation of the HSP73 from HSP72 forms of HSP70. *P*-values were obtained using a two-way analysis of variance (ANOVA) followed by Tukey’s multiple comparisons test.

**Figure 5:**
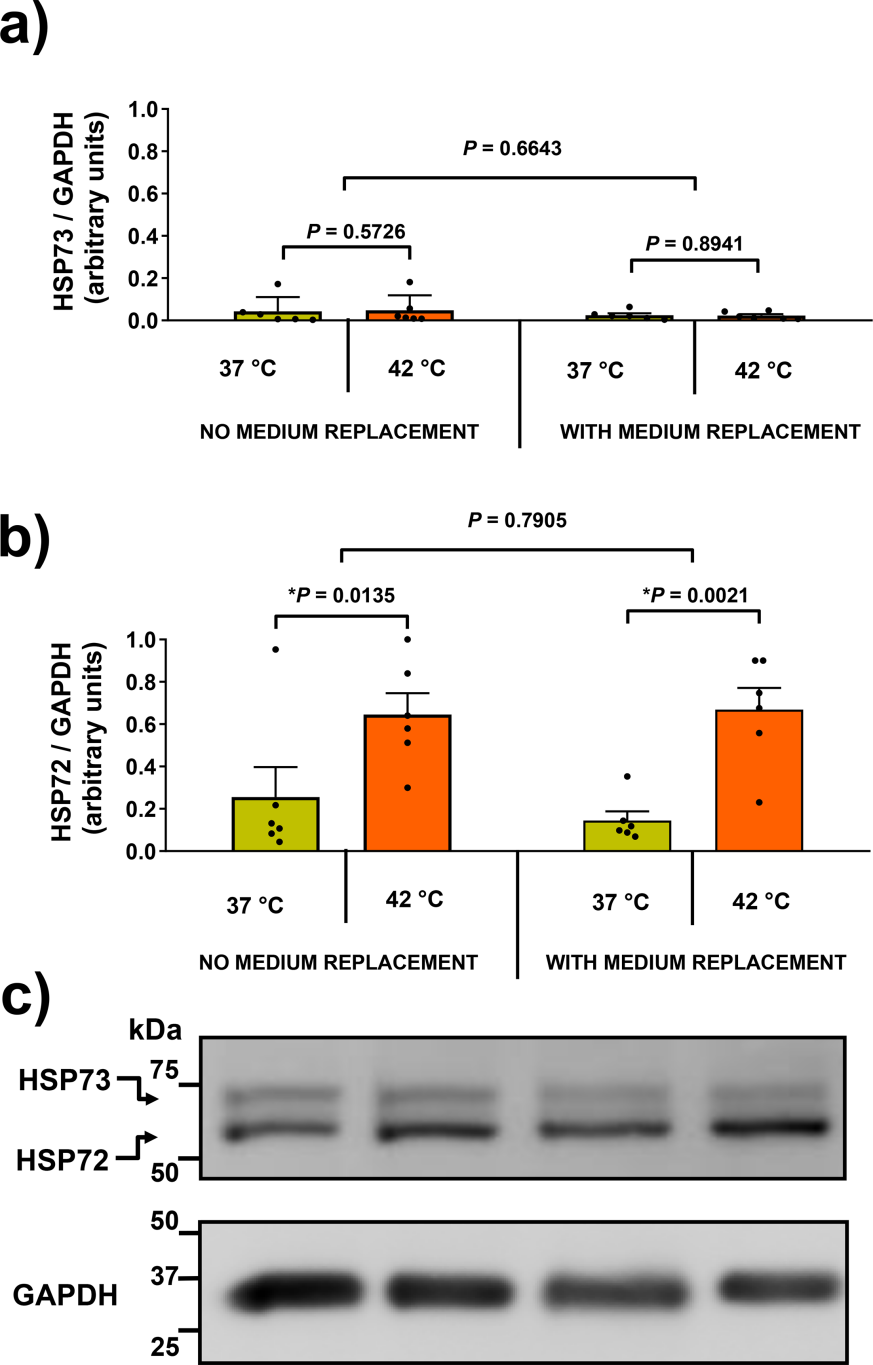
Impact of medium replacement on HSP70 expression in whole-blood preparations. This figure illustrates the influence of medium replacement on HSP70 expressions in heat-challenged whole-blood samples. Blood samples were collected from adult male mice after an overnight fast (*n*=12). The mice were divided into two groups: one with medium replacement (*n*=6) and the other without (*n*=6). The immunocontents of **(a)** ‘cognate/constitutive’ HSP73 (hspa8) and **(b)** ‘inducible’ HSP72 (hspa1a and hspa1b) forms of HSP70 in whole-blood samples were assessed. These samples were prepared as described in the legend of Figure 2, with one difference: in half of the samples, the culture medium was changed after a 2-hour heat (or control) treatment, followed by a 6-hour recovery period at 37°C. The remaining samples were maintained without medium replacement. Representative gels **(c)** provide a visual representation of the separation between the HSP73 and HSP72 forms of HSP70. The expressions, relative to GAPDH (western blotting), are presented as means ± S.D. (*n*=6) for each lane. Statistical significance, indicated by the given *P*-values, was determined using a two-way analysis to variance (ANOVA) followed by Tukey’s multiple comparisons test.

In response to the heat challenge, both male and female mice exhibited unaltered HSP73 (hspa8 gene product) expression (Figure 4a). However, a significant increase (approx. 50 %–75 %) in HSP72 (hspa1a and hspa1b gene products) was observed after 2-hour incubations, but not after 1 hour, in both sexes (Figure 4b). Tests were also done to compare HSP72 release in fasted versus fed animals, considering the inhibitory effect of elevated glycaemia [82]. Feeding animals extinguished the HSR, evidenced by diminished HSP72 expression in both male and female samples (Figure 4b). Subsequent experiments involving fasted male mice and culture medium replacement after the 2-hour heat challenge demonstrated that heat-induced HSP72 expression remained unaffected (Figure 5b). In this case, HSP73 expression consistently exhibited no response to heat treatment (Figure 5a).

**Figure 6:**
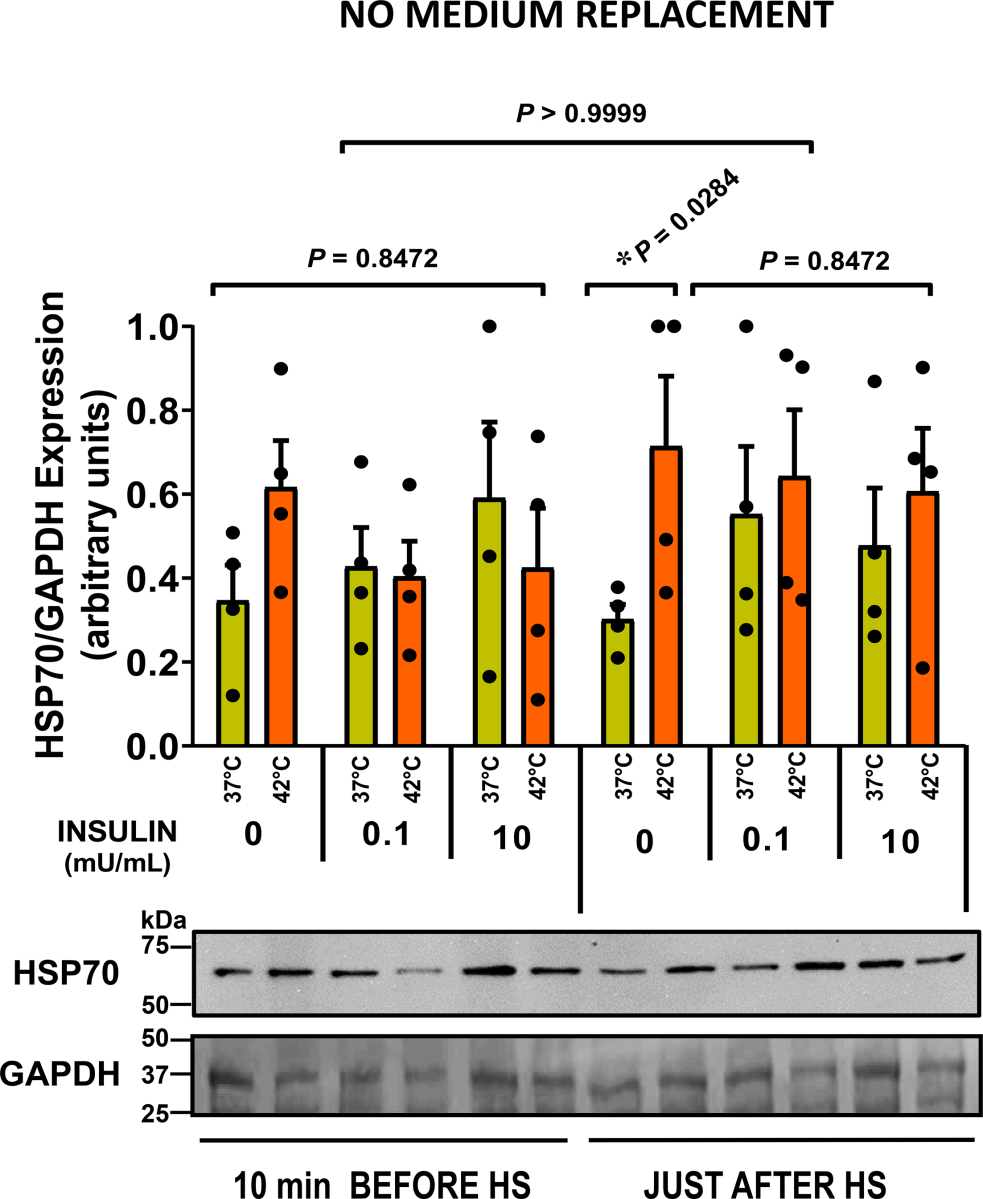
Influence of insulin on HSP70 expression in heat-challenged whole blood samples from adult male rats. Shown is the effect of insulin on HSP70 (HSP72+HSP73) expression in whole-blood samples obtained from 60-day-old male rats after an overnight fast (*n*=4). The samples were prepared following the protocol described in the legend of Figure 2. These samples underwent a 2-hour heat challenge at either 42 °C or 37 °C in the presence or absence of maximal physiological (0.1 mU/mL) or pharmacological (10 mU/mL) levels of insulin. Insulin was introduced either 10 min before the heat treatment (or control) or immediately after the heat shock (HS) period. Subsequently, the samples were incubated for an additional 6 hours without medium replacement, and western blot analysis was used to calculate mean expressions (means ± S.D., *n*=4) for each lane. It is worth noting that in rat cells, HSP73 and HSP72 do not separate as distinctly as they do in mouse cells, even when employing the same running conditions and the same monoclonal antibodies (Sigma H5147, clone BRM22 ascites fluid). Statistical differences, denoted by the provided *p*-values, were assessed through a two-way analysis of variance (ANOVA) and Tukey’s multiple comparisons test. The figure includes a representative gel with samples from one single animal, with the loading control (GAPDH) determined from a Red Ponceau S-stained membrane.

Since insulin regulates HSP70 expression [89], it is conceivable that insulin could have contributed to the outcomes observed in the fed-animal samples depicted in Figure 4b. Insulin’s role in HSP70 expression was investigated using fasted male rat blood. Incubation with post-prandial (100 μU/mL) or supraphysiological (10 mU/mL) insulin doses showed no discernible HSP70 alterations before the heat challenge (Figure 6). However, the introduction of insulin after the 2-hour heat challenge, sustained during recovery, negated the substantial increase in HSP72 expression. Insulin possibly influences basal HSP72 expression, showing an 83 % increase at 0.1 mU/mL and 58 % at 10 mU/mL, though statistically nonsignificant (*P*=0.8904 and *P*=0.9405, respectively). This could explain the lack of significant differences in just-after heat-shock insulin groups between 37 °C and 42 °C groups (*P*=0.8472).

**Figure 7:**
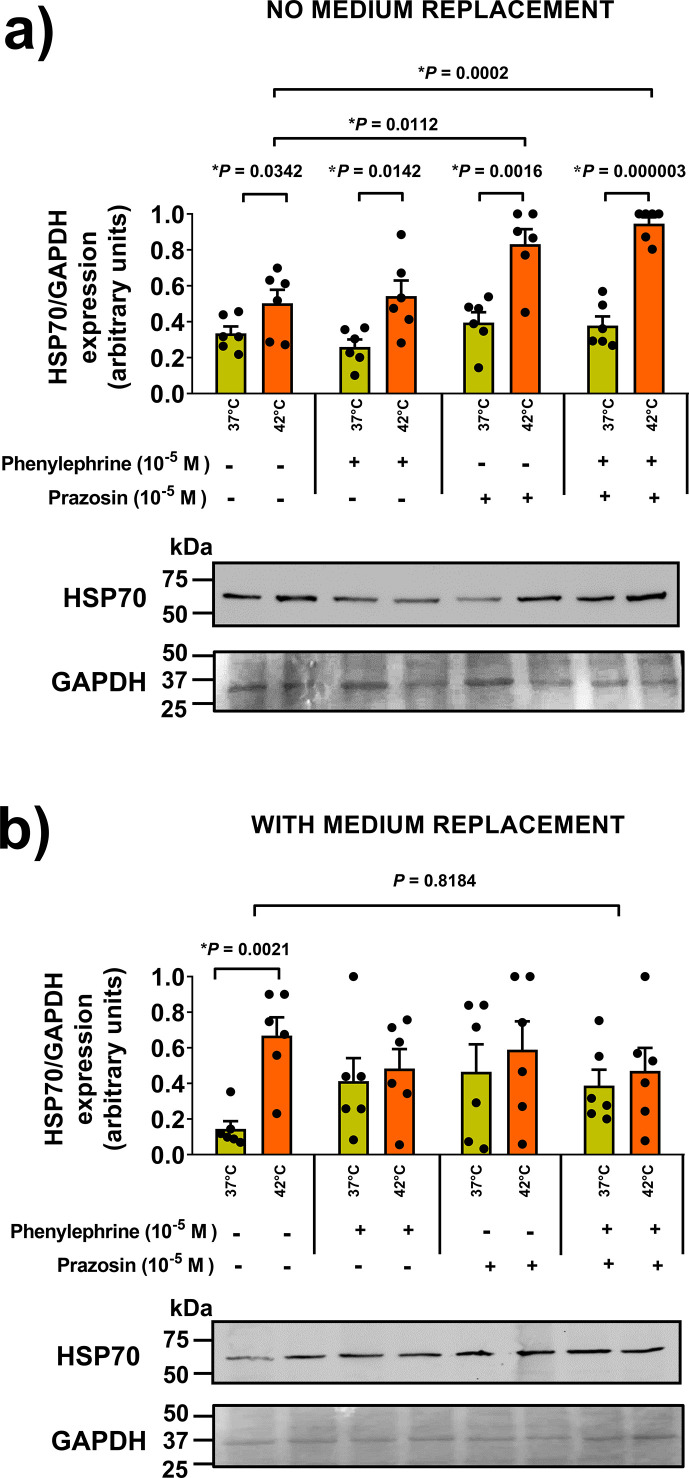
Repercussions of α_1_-adrenergic stimulation (or blockade) on HSP70 (HSP72+HSP73) expression in heat-challenged whole-blood samples from adult male rats. Whole-blood samples were obtained from overnight-fasted adult male rats (60 days old; *n*=6). The samples were prepared according to the protocol described in the legend of Figure 2. Half of the samples underwent a 2-hour heat treatment at 42 °C (or a control at 37 °C), followed by a 6-hour recovery period at 37 °C, with a culture medium change occurring after the heat treatment. The remaining samples were maintained without medium replacement. To examine the influence of α_1_-adrenergic stimulation, samples were treated (or not) with 10^-5^ M phenylephrine, and/or the α_1_-blocker, 10^−5^ M prazosin, as detailed in the illustration. The drugs were added at the beginning of the 2-hour heat challenge (or control) test and were either maintained (or re-added) throughout the entire 8-hour experiment for the no-replacement medium study **(a)**. For the replaced medium samples **(b)**, the drugs were present only during the first 2-hour period before the medium change. Mean expressions (mean ± S.D., *n*=6) for each lane were calculated from western blot analysis. As previously mentioned in the legend for Figure 6, it is important to note that HSP73 and HSP72 do not separate as distinctly in rat cells as they do in mouse cells, even when employing the same experimental conditions. Statistical differences, indicated by the provided *p*-values, were assessed through a two-way analysis of variance (ANOVA) and Tukey’s multiple comparisons test. The figure includes representative gels, each containing samples from a single animal, with the loading control (GAPDH) determined from a Red Ponceau S-stained membrane.

In the PBL pool, lymphocytes contribute almost 100% to HSP72 synthesis *in vitro* [49], regulated by the α_1_ adrenergic receptor *in vivo* [90]. Therefore, investigating metabolic stress during fasting (adrenergic response), we treated blood from starved rats with phenylephrine or phenylephrine plus prazosin (specific α_1_-blocker), maintaining the drugs throughout the experiment. Phenylephrine showed no impact on HSP72 expression (Figure 7a), while prazosin alone increased it by 65 % (*P*=0.0112) compared with the control 42 °C group (*cf*. Figure 7a and b). Even in the presence of phenylephrine, the prazosin-induced increase was more pronounced (88 %, *P*=0.0002), suggesting autocrine catecholamine suppression of HSP72, as seen in cell proliferation and cytokine production [91,92]. Although HSP72 expression in response to heat stress was unaffected by medium change (*P*=0.1381), the absence of either phenylephrine or its combination with prazosin in the culture medium after heat stress (Figure 7b) reversed the effects observed in Figure 7a.

### Dietary impact on body parameters in male mice

**Figure 8:**
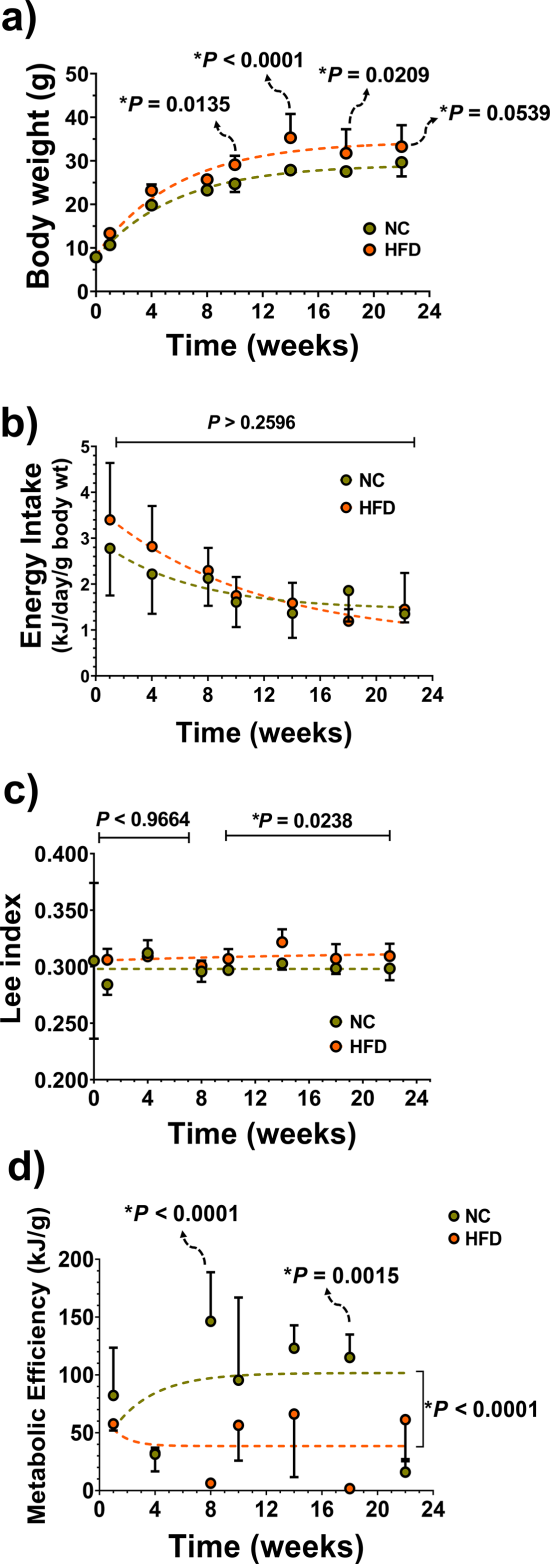
Dietary impact on body parameters in male mice. Evolution of body weight **(a)**, energy intake **(b)**, ‘nutritive’ Lee index **(c)** and metabolic efficiency **(d)** in male mice fed either a high-fat diet (HFD; orange symbols and lines, *n*=6 per time point) or standard normal chow (NC; green symbols and lines, *n*=6 per time point). Mice were weaned at 21 days of age and randomly assigned to their respective diets for a duration of up to 22 weeks. Dashed lines represent non-linear regressions for both treatments in all analyses. Statistical differences were assessed through mixed-effects analysis and Sidak’s multiple comparisons test. Multiplicity-adjusted *P*-values for the comparisons between NC and HFD groups at each time point are appropriately displayed. Specifically for the Lee index **(c)**, mixed-effects analysis revealed distinct behaviours between NC and HFD after 8 weeks of diet intervention. Consequently, unpaired two-tailed multiple *t*-tests were conducted, considering two time intervals: from T0 to T8 and from T10 to T22. As a result, two *P*-values are presented, one for each time interval. For all measurements, data are presented as mean ± S.D.

As illustrated in Figure 8a, HFD led to a substantial increase in body weight after 10 weeks on the regimen compared with controls. Notably, energy intake remained consistent in both groups throughout the experimental period, as depicted in Figure 8b. After 8 weeks on the HFD, the nutritive Lee index was elevated in HFD animals compared with controls, as shown in Figure 8c. Simultaneously, the metabolic efficiency of HFD animals began to decline (*P*<0.0001) after 8 weeks on the diet, as evidenced in Figure 8d.

In the current HFD model, it is observed a trend of decreasing energy intake while maintaining a consistent difference between the control and test groups, as previously reported [27,44]. Because of this, we calculated a parameter known as metabolic efficiency [27,44], reflecting the body’s ability to metabolise ingested energy. Metabolic efficiency was determined by dividing the energy intake by the body weight gain weekly. During the rapid growth phase, energy intake remains stable, but metabolic efficiency exhibits an increasing trend over time for both NC and HFD groups, indicating that ingested energy is less likely to result in body weight gain. Notably, however, the metabolic efficiency index is lower in HFD-fed mice in late stages compared with those on an NC diet, serving as an inverse measure of feed efficiency (i.e., weight gain per unit of ingested energy), a parameter previously found to be elevated in HFD-fed mice [93]. As suggested by Winzell and Ahrén [44], this observation implies that the weight gain observed in HFD-fed mice cannot be solely attributed to increased energy intake but is also influenced by a reduced metabolic rate. This remark is crucial for the understanding of the evolution of the anti-inflammatory HSR because the HSR is intertwined with energy metabolism through the AMPK and HSF1 pathways (please see Figure 1).

### Evolution of the HSR to heat challenge

**Figure 9:**
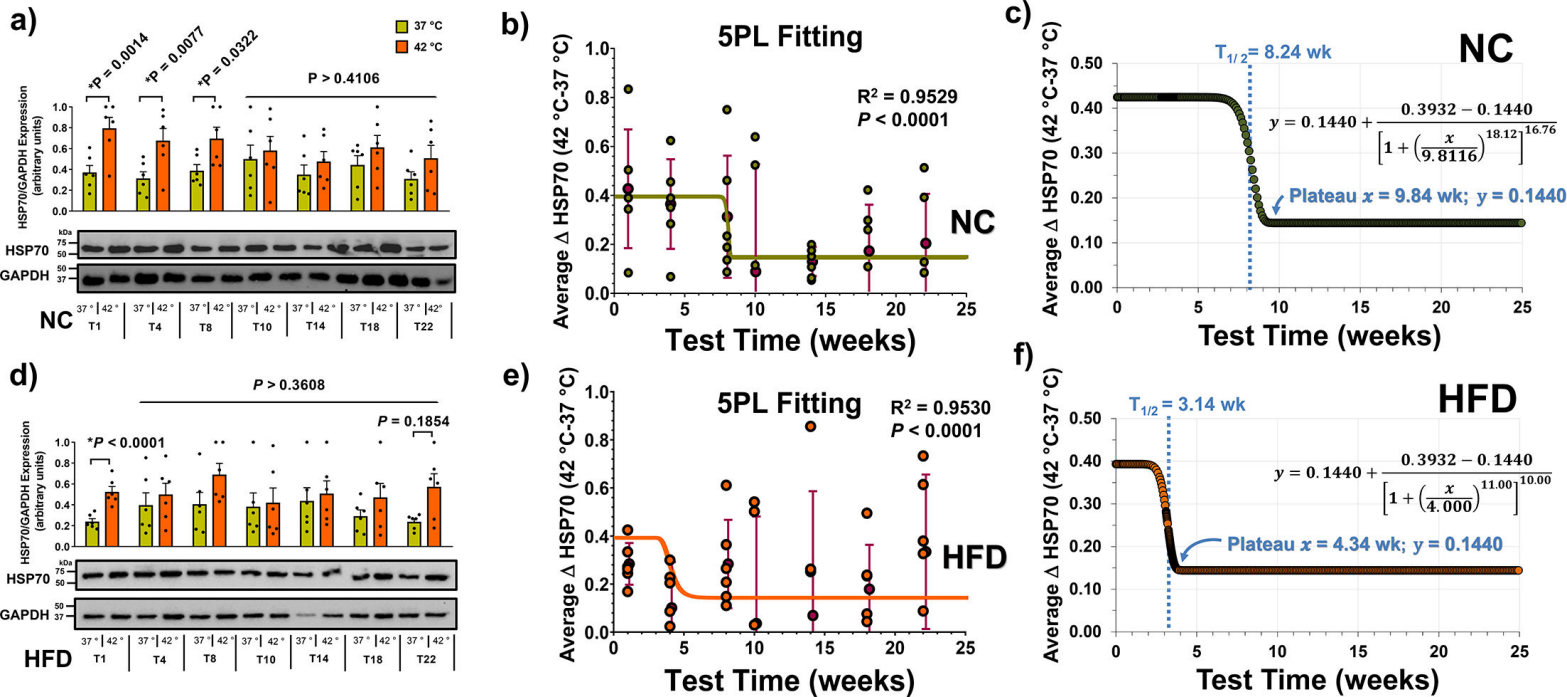
Time course of heat-induced HSP70 expression in whole blood of male mice under high-fat diet (HFD) or standard normal chow (NC). **(a)** The histogram depicts HSP70 expressions relative to GAPDH following whole-blood incubations at 37 °C (green) or 42 °C (orange) across T1 to T22 weeks for the **NC** group (*n*=6 per time point). Representative gels below the histogram illustrate the differential expressions. Statistical comparisons between control and 42 °C challenging groups were conducted using two-way analysis of variance (ANOVA) and Sidak’s multiple comparisons test, yielding adjusted *P*-values. **(b)** Heat shock response (HSR) over time. The HSR, expressed as ΔHSP70 [i.e., ΔHSP70 = (HSP70 at 42 °C) – (HSP70 at 37 °C)], is plotted against time points (T1–T22) using an asymmetric five-parameter logistic (5PL) regression. The green line represents the average ΔHSP70, while individual data points (green balls) and means ± S.D. (lilac balls-and-bars) are displayed. Least squares fit was employed, and both *P*-values and *R*^2^ were calculated as described in the Methods section. **(c)** Predicted ΔHSP70 over time. The 5PL-regression function predicts ΔHSP70 as a function of time (equation at the centre of the panel). Key parameters such as inflection time (*T*_1/2_) and plateau values (***x* and *y***) are provided. Panels **(d)**, **(e)** and **(f)**: HSP70 expressions in **HFD** mice. Similar to the NC groups, panels **(d)**, **(e)** and **(f)** show, respectively, HSP70 expressions, the HSR (ΔHSP70; orange line and balls) and predicted ΔHSP70 over time in HFD mice. Lilac balls-and-bars indicate means ± S.D. (*n*=6 per time point). Statistical analyses and predictions mirror those described for NC groups in panels **(a)**, **(b)** and **(c)**.

Whole-blood samples from fasted male mice on a control NC diet exhibited the expected increase in HSP72 expression up to the eighth week, but beyond this, the ability to respond to heat challenges by increasing HSP72 was lost (Figure 9a). Conversely, HFD mice responded only in the first week (Figure 9d). The HSR, expressed as the average ΔHSP70 [i.e., ΔHSP70 = (HSP70 at 42 °C) – (HSP70 at 37 °C)], was modelled using a 5PL regression (Figure 9b and e). Predicted values revealed an inflection point (*t*_1/2_) at 8.24 weeks for the NC group and 3.14 weeks for the HFD group (Figure 9c and f). This suggests an earlier loss of heat-induced HSR capacity in the HFD group, while control animals surprisingly lost it before the midpoint of the 22-week experimental period.

### Evolution of the glycaemic status

**Figure 10:**
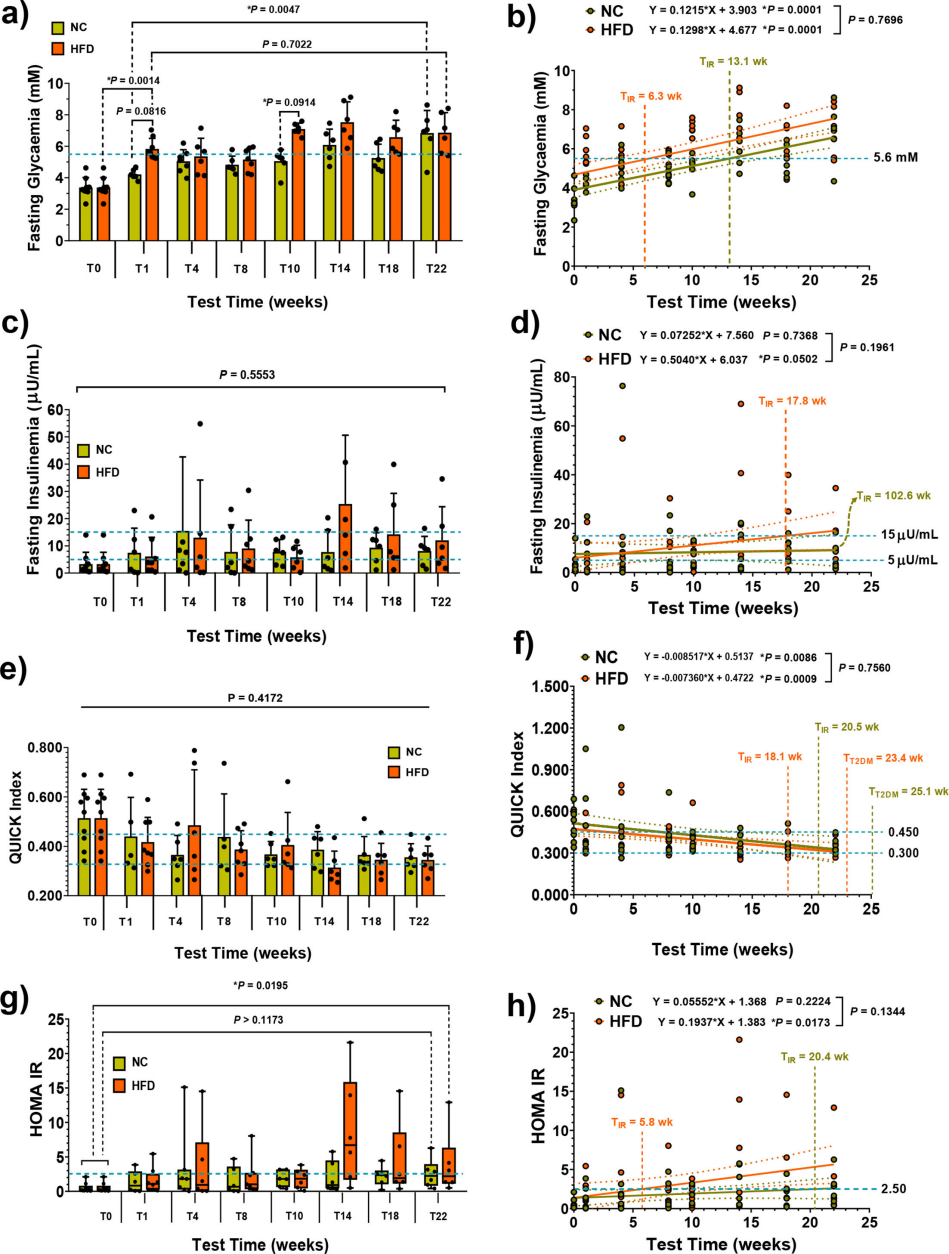
Glycaemic status evolution in male mice under standard normal chow (NC; green symbols) or high-fat diet (HFD; orange symbols). **(a)** This panel illustrates the time course of fasting glycaemia from weaning (**T0**) to week 22 (**T22**). Caerulean dashed lines represent the expected normal fasting glycaemia (5.6 mM). **(b)** Linear regression equations derived from the data in panel **(a)** are presented alongside *P*-values for each curve and for the difference between NC and HFD curves. Dotted lines around solid regression lines (green for NC and orange for HFD) indicate the 95 % confidence interval (95% CI) for each function. Putative test times for the onset of impaired glucose tolerance (IGT) or insulin resistance (fasting glycaemia above 5.6 mM) are provided for both diet groups (T_IR_). **(c)** The time course of fasting insulinaemia is presented, along with linear regressions and colour representations consistent with panels **(a)** and **(b)**. Caerulean dashed lines define the physiological range of expected fasting insulinaemia (5–15 μU/mL), while expected insulin resistance times (T_IR_) are indicated in weeks **(d)**. Panels **(e)** and **(f)** display the time course of QUICK index (QUICKI) values and respective linear regressions with colour representations consistent with previous panels. The normal range of QUICKI values and putative times for the onset of insulin resistance (T_IR_; below 0.450) and the expected onset of type-2 diabetes mellitus (T_T2DM_; below 0.300) are shown. **(g)** Time course of HOMA-IR index values and linear regressions are depicted, with colour schemes consistent with earlier panels. Normal HOMA-IR values (up to 2.50) and putative times for the onset of insulin resistance (T_IR_) are provided. Statistical differences between NC and HFD groups for fasting glycaemia, fasting insulinaemia, QUICKI and HOMA-IR were assessed through mixed-effects analysis and Tukey’s multiple comparisons test. Multiplicity-adjusted *P*-values for the comparisons between NC and HFD groups at each time point are displayed. Histograms (**a**, **c** and **e**) present means ± S.D., whereas data in the histogram **(g)** represent the median, maxima, minima and the 95% CI. In all cases, *n*=6 per time point.

Fasting glycaemia in HFD animals remained around 5.6 mM until week 8 but rose to approximately 7 mM from week 10 onwards (Figure 10a). Although the slopes of the NC and HFD curves were indistinguishable (*P*=0.7696; Figure 10b), they had different y-intercepts on the glycaemic axis. Considering values above 5.6 mM as indicative of impaired fasting glycaemia (IFG) [94,95] or inceptive IR, we plotted the respective individual values as a function of test time, revealing an IR onset time (*T*_IR_) of 6.3 weeks for HFD animals and 13.1 weeks for the controls (Figure 10b). Both groups surpassed the critical threshold for type-2 diabetes onset during the experimental period (T22 weeks for NC and T14 weeks for HFD). Fasting insulinaemia did not differ significantly (Figure 10c), but HFD animals intersected with insulinaemia values surpassing 15 μU/mL at 17.8 weeks, while controls would do so at 102.6 weeks (Figure 10d). QUICK index and HOMA-IR values showed minimal differences, but HFD animals crossed the HOMA-IR limit earlier at 5.8 weeks compared with NC mice, at 20.4 weeks (Figure 10e–10h).

### Correlation between glycaemic status and the HSR

**Figure 11:**
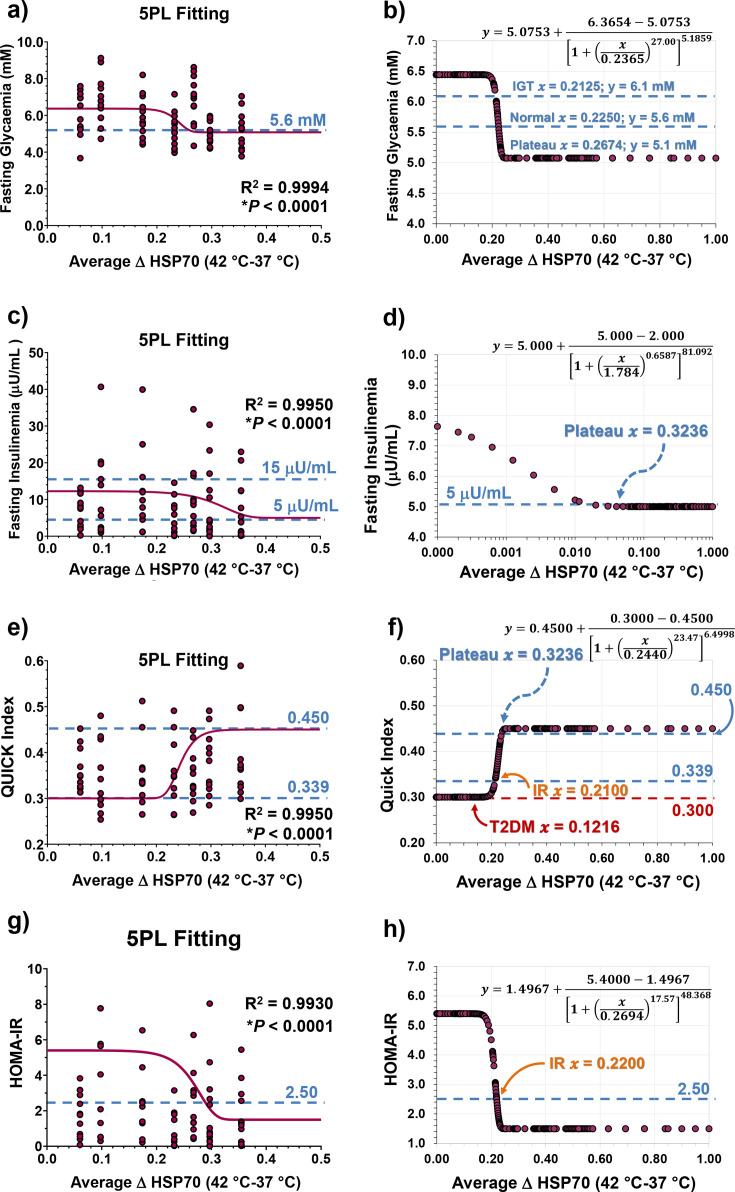
Glycaemic status and heat shock response (HSR) parallelism in male mice. This figure establishes a parallel between glycaemic status and the HSR evaluated through whole-blood heat challenge-induced HSP70 expressions in male mice. Values for average ΔHSP70 [ΔHSP70 = (HSP70 at 42 °C) – (HSP70 at 37 °C)] shown on the abscissae of the panels were computed from the time course of ΔHSP70 data presented in Figure 9a (NC; *n*=6) and Figure 9d (HFD; *n*=6), irrespective of diet regimen. The average was calculated as an overall mean for each time point (*n*=12). Subsequently, the glycaemic status indicators (lilac balls) from Figure 10 were plotted against the mean ΔHSP70 values for the same time points (T1–T22). This was achieved using an asymmetric five-parameter logistic (5PL) regression, represented by lilac solid lines in panels (**a)**,**(c)**,**(e)** and **(g)**. Predicted glycaemic status parameters as a function of ΔHSP70 by 5PL-regression functions (equations at the upper part of the panels) are given in panels **(b)**,**(d)**,**(f)** and **(h)**. Caerulean dashed lines in the panels represent the expected normal values/ranges for fasting glycaemia, fasting insulinaemia, QUICKI and HOMA-IR. For fasting glycaemia, normal values (below 5.6 mM) and the interval between impaired fasting glycaemia (IFG; ≥5.6 and <6.1 mM) and impaired glucose tolerance (IGT; 6.1 mM) are given. Key parameters, including plateau values (*x*) and expected ΔHSP70 values for the onset of insulin resistance (IR) and type-2 diabetes mellitus (T2DM) from the reconstructed regression curves, are also provided. Least squares fit was employed for the analysis, and both *P*-values and *R*^2^ were calculated as described in the Methods section. Note that the ΔHSP70 fit to fasting insulinaemia is presented in a logarithmic scale (**d**).

When consolidating the comprehensive results, as illustrated in Figure 9 (evolution of heat-induced HSR) and Figure 10 (glycaemic status over time), a 5PL regression model was applied, resulting in Figure 11. The average ΔHSP70 [ΔHSP70 = (HSP70 at 42 °C) – (HSP70 at 37 °C)] values on the abscissae were computed from the time course of ΔHSP70 data presented in Figure 9a (NC) and Figure 9d (HFD), regardless of the diet regimen — calculated as an overall mean for each time point. Fasting glycaemia values were graphed against ΔHSP70 values (Figure 11a). The reconstructed 5PL curve reveals a correlation, with ΔHSP70 values surpassing 0.2674 corresponding to fasting glycaemia below 5.1 mM (Figure 11b). Furthermore, the predicted ΔHSP70 value for normal fasting glycaemia (<5.6 mM) was determined to be 0.2250, and for IFG (glycaemia ≥ 5.6 and <6.1 mM), it falls within the range of 0.2250–0.2125. Values lower than 0.2125 indicate the onset of type-2 diabetes (fasting glycaemia ≥6.1 mM). The same approach was taken for fasting insulinaemia (Figure 11c), revealing that ΔHSP70>0.3236 correlated with fasting insulinaemia below 5 μU/mL (Figure 11d). In terms of QUICKI correlations (Figure 11e), the reconstructed curve supports that ΔHSP70 values between 0.3236 and 0.2100 are predicted to be in the normal range, while values between 0.2100 and 0.1216 suggest IR, and those below 0.1216 indicate the onset of type-2 diabetes (Figure 11f). Regarding HOMA-IR (Figure 11g), the reconstructed curve suggests the onset of IR when ΔHSP70 values fall below the 0.2200 mark (Figure 11h).

**Figure 12:**
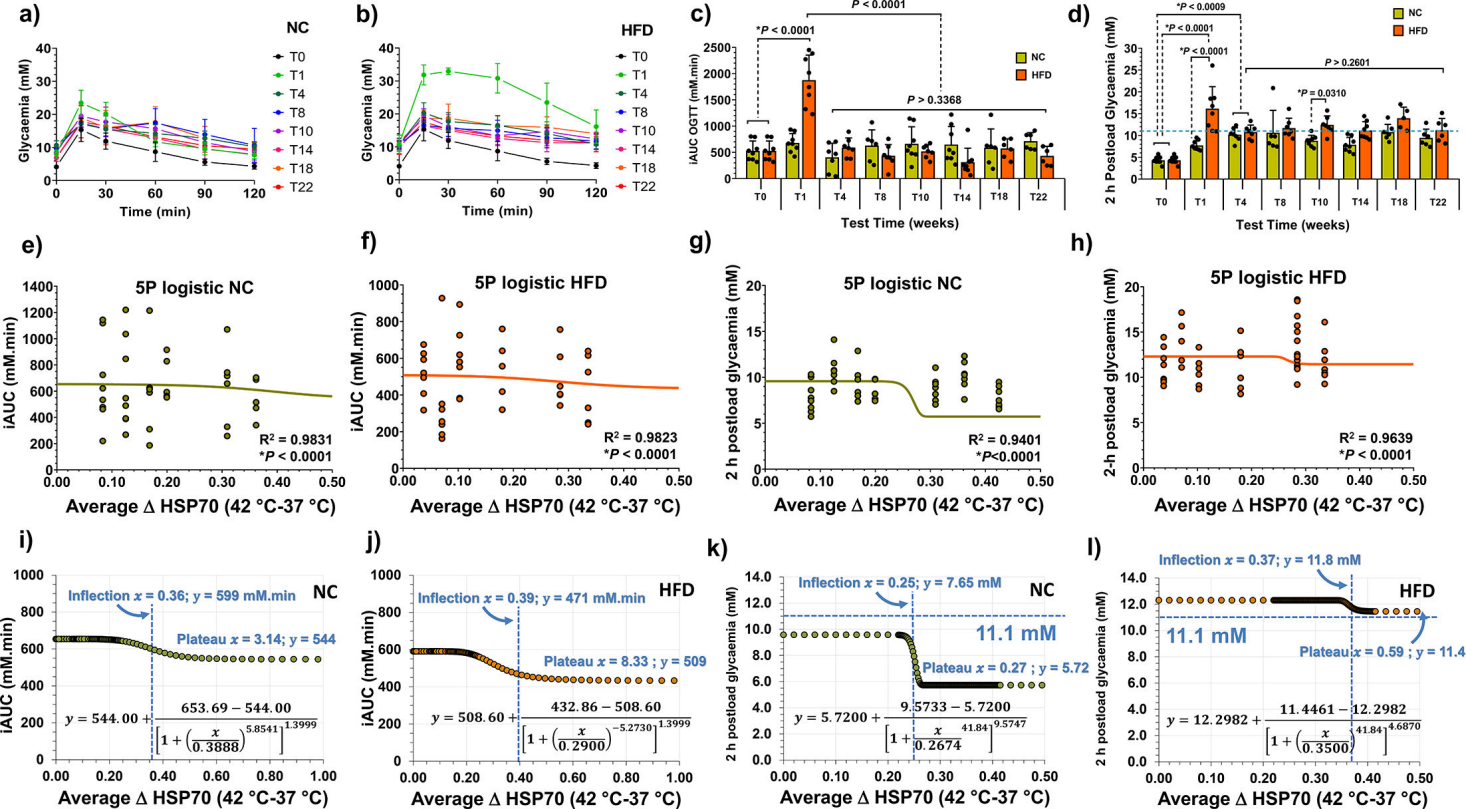
Comparative analysis of oral glucose tolerance tests (oGTT) and heat shock response (HSR) in fasted male mice. The curves depict the behaviour of oGTT across experimental time points (from T0 to T22 weeks) for male mice under normal standard chow (NC; panel **(a)** and high-fat diet (HFD; panel **(b)**, while data in the curves are presented as the means ± S.D. (*n*=8 per group). For oGTT, statistical significance was assessed by repeated measures analysis of variance (ANOVA) followed by Tukey’s multiple comparisons test (online Supplementary figure 1). **Glycaemic status**: Panel **(c)** displays the evolution of incremental areas under glycaemic curves (iAUC) for both NC (green) and HFD (orange) groups throughout the experimental period. Panel **(d)** presents the 2-hour post-glucose load glycaemia for the same groups. Histograms in panels **(c)** and **(d)** show means ± S.D. (*n*=8 per group). Statistical comparisons between NC and HFD groups employed two-way ANOVA and Sidak’s multiple comparisons test, with multiplicity-adjusted *P*-values provided in the respective panels. **Integration with the HSR:** In alignment with the methodology described in the legend of Figure 11, iAUC values were plotted against average ΔHSP70 (an indicator of HSR magnitude) for the corresponding time points, except for T0 animals (see Sample Size section). The resulting five-parameter logistic (5PL) regression for NC (solid green line) is presented in panel **(e)** alongside individual values (green balls), while the same is illustrated for the HFD group in panel **(f)** (orange line and balls). **Correlation of postload glycaemic values with ΔHSP70**: Panels **(g)** and **(h)** depict 5PL regressions between 2-hour postload glycaemic values and ΔHSP70 for NC and HFD, respectively, using the same colour representations as above. **Reconstructed regression curves**: Panels **(i)**, **(j)**, **(k)** and **(l)** showcase reconstructed regression curves obtained from the 5PL functions of panels **(e)**, **(f)**, **(g)** and **(h)**. Equations (lower part of the panels) and key parameters, including plateau values (***x***) and inflection points (***x* and *y***), are provided with cerulean dashed lines. Least squares fit was employed for the analysis, and both *P*-values and *R*^2^ were calculated as described in the Methods section. Horizontal dashed cerulean lines in panels **(d)**, **(k)** and **(l)** represent the upper limit of impaired glucose tolerance (IGT) in 2-hours postload glycaemia (11.1 mM), while vertical lines are the inflection points.

To assess glucose tolerance and type-2 diabetes onset, oGTTs were conducted (Figure 12a and b). After just 1 week on the HFD, the iAUC rose remarkably, 177 % higher than NC controls (Figure 12c). After this point, iAUC values aligned with T0 values, persisting until the study’s end. HFD animals showed a 279 % surge in 2-hour post-load glycaemia after just 1 week, while NC animals had a 79 % increase. From 4 weeks onwards, both groups maintained 2-hour postload glycaemia around the upper limit of IGT (≥7.8 and <11.1 mM) and type-2 diabetes thresholds (≥11.1 mM). 5PL correlation analysis with average ΔHSP70 values (Figure 9a and d) revealed that ΔHSP70 above 0.36 (NC) and 0.39 (HFD) corresponded to normal glycaemic range (Figure 12i and j). Similarly, the reconstructed 5PL curves also exposed a robust correlation between 2-hour postload glycaemia and average HSP70, with ΔHSP70 values above 0.25 corresponding to 2-hour postload glycaemia between 5.72 mM and 7.65 mM (normal range) for NC animals (Figure 12k). In contrast, for HFD animals (Figure 12l), ΔHSP70 values above 0.37 corresponded to a 2-hour postload glycaemia between 11.4 mM (IGT) and 11.8 mM (type-2 diabetes mellitus), implying that HFD mice, at all time points, exhibit IR or are diabetic according to the established ΔHSP70 criterion after the first week on the diet.

**Figure 13:**
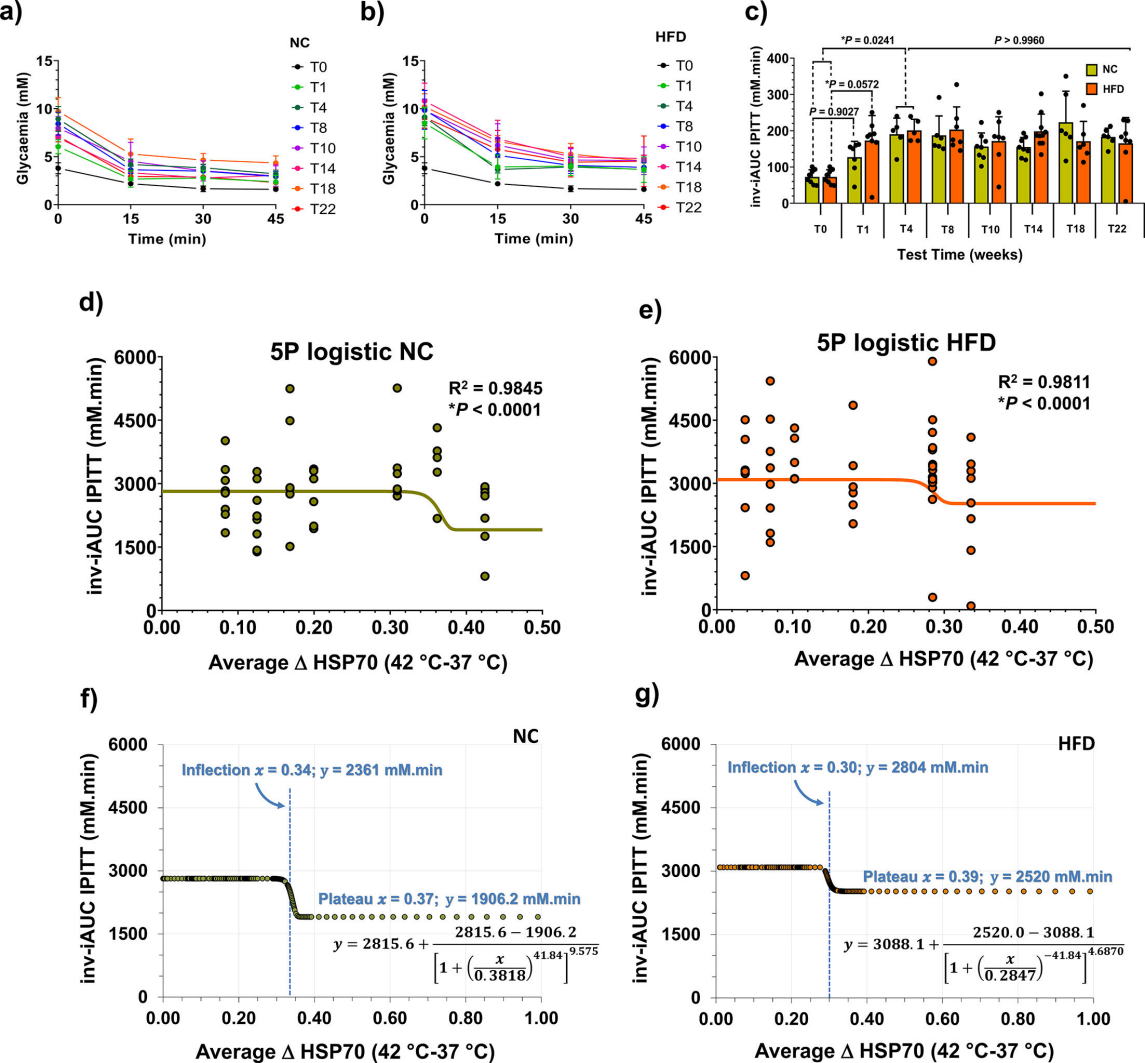
Comparative analysis of intraperitoneal insulin tolerance tests (ipITTs) and the heat shock response (HSR) in male mice. The curves depict the behaviour of ipITT across experimental time points (from T0 to T22 weeks) for male mice under normal standard chow (NC; panel **(a))** and high-fat diet (HFD; panel **(b))**, whereas data in the curves are presented as the means ± S.D. (*n*=8 per group). For ipITT, statistical significance was assessed by repeated measures analysis of variance (ANOVA) followed by Tukey’s multiple comparisons test (please, see Supplementary figure 1). **Glycaemic status**: Panel **(c)** displays the evolution of inverted incremental areas under glycaemic curves (inv-iAUC) for both NC (green) and HFD (orange) groups throughout the experimental period. Histograms in panel **(c)** depict means ± S.D. Statistical comparisons between NC and HFD groups utilised two-way ANOVA and Sidak’s multiple comparisons test, with multiplicity-adjusted *P*-values provided. **Integration with the HSR:** Following the methodology described in the legends of Figures 11 and 12, inv-iAUC values were plotted against average ΔHSP70, an indicator of HSR magnitude. T0 animals were excluded, as explained in the Sample Size section. These data are fitted with five-parameter logistic (5PL) regressions, and the resulting 5PL functions for NC (solid green line) and HFD (solid orange line) are presented in panels **(d)** and **(e)** alongside individual values. **Reconstructed regression curves**: Panels **(f)** and **(g)** showcase reconstructed regression curves obtained from the 5PL functions of panels **(d)** and **(e)**. Equations and key parameters, including plateau values (***x***) and inflection points (***x* and *y***), are provided with cerulean dashed lines. Least squares fit was employed for the analysis, with both *P*-values and *R*^2^ calculated following the techniques outlined in the Methods section.

ipITTs were conducted (Figure 13a and b). After just 1 week, both NC and HFD groups showed significantly elevated inv-iAUC, with a 97.4 % increase for NC and a staggering 138 % rise for HFD compared with T0 values (Figure 13c). This elevation persisted until week 4 on both diets, with a consistent upward slope (161 % increase for NC, 167 % for HFD). Values for both groups remained elevated from week 4 onwards until the study’s end. Correlation analysis with average ΔHSP70 values (Figure 9a and d) showed ΔHSP70 above 0.34 (NC) and 0.30 (HFD) corresponded to lower inv-iAUC values for ipITT (Figure 13d and e). Although not aligning with established IR surrogates, plateau values for HFD animals (2,804 mM.min) were notably higher than NC mice (1906.2 mM.min), suggesting a tendency towards IR in HFD animals (Figure 13f and g).

## Discussion

Through *ex vivo* heat challenge experiments on whole blood samples from both NC and HFD mice, we established a 5PL correlation between the HSR and experimental time, as measured by ΔHSP70. Our findings revealed a progressive loss of HSR in both groups (Figure 9), with HFD mice exhibiting a significantly earlier decline (*t*_1/2_ = 3.14 weeks) compared with NC mice (*t*_1/2_ = 8.24 weeks). This indicates compromised HSR-mediated anti-inflammatory capacity in HFD mice, but control animals were also affected. Notably, whole-blood heat challenge proved equally effective in male and female animals, as long as they were fasted, and responses were unaffected by medium replacement post-thermal treatment. Interestingly, physiological insulin doses were found to enhance basal HSP70 expression, preventing the heat-induced rise in HSP70. This may explain the lack of response to heat challenge in fed animals, as adrenergic participation in fasting animals was inconclusive (Figure 7).

The decline in ΔHSP70 over time correlated with unfavourable glycaemic status. The 5PL correlation revealed that ΔHSP70 values above 0.2250 corresponded to fasting glycaemia below the expected normal limit of up to 5.6 mM (Figure 11). In contrast, ΔHSP70 in the range of 0.2125–0.2250 indicated IFG (≥5.6 and <6.1 mM), with ΔHSP70 of 0.2150 corresponding to IGT (6.1 mM). ΔHSP70 below 0.2125 signalled the onset of type-2 diabetes [94,95]. Additionally, ΔHSP70 higher than 0.2500 corresponded to a 2-hour postload glycaemia lower than 7.65 mM (normal) in NC mice (Figure 12), but not in HFD animals, suggesting the onset of type-2 diabetes in the latter. Moreover, ΔHSP70 higher than 0.2200 correlated with HOMA-IR values lower than 2.50, the maximum expected value prior to IR (Figure 11), while ΔHSP70 higher than 0.2100 indicated a QUICKI within the normal range (0.339–0.450). ΔHSP70 between 0.1216 and 0.2100 corresponded to QUICKI in the range of IR (0.300–0.339), and below 0.1216 indicated the onset of type-2 diabetes. Overall, results suggest normal glycaemic status when ΔHSP70 is above 0.22, and IR may be expected when ΔHSP70 is below 0.21.

It is noteworthy that, as shown in Figure 9c, the heat-induced increase in HSP70 expression begins to diminish by week (*t*_1/2_) 8.24 in control animals and even earlier, at 3.14 weeks, in HFD mice. Notably, this decline in heat-induced HSP70 expression precedes the onset of IFG, which itself emerges before IR, as illustrated in Figure 10b. Thus, changes in HSP70 (ΔHSP70) may serve as an early marker of disrupted glycaemic status, appearing well before any prodromal signs of IR, which become evident in both groups between weeks 18.1 and 20.5 based on QUICKI results (Figure 10f). Overall, these findings suggest that ΔHSP70 indicates early glycaemic dysregulation, even when fasting glycaemia remains within the normal range, maintained through homeostatic adjustments involving insulin and counter-regulatory mechanisms in adipose tissue, liver and skeletal muscle [74,94,95].

Notably, the temporal decrease in ΔHSP70 aligned with the emergence of weight gain, Lee index elevation and decreased metabolic efficiency in HFD animals (Figure 8), indicating an association between obesity and ΔHSP70 findings. This is significant as suppressed expression of both HSP70 and HSF1 is commonly observed in adipose tissue, liver, skeletal muscle and vascular beds of individuals with obesity and type-2 diabetes mellitus [1,3,8,11–18,24,34]. Furthermore, HSR failure in obesity is linked to increased low-grade inflammation, including activation of JNKs [16,96,97] and NLRP3 inflammasome persistence [98–100], where HSP70 serves as a negative regulator of both JNKs [24] and NLRP3 inflammasome activation [101]. Hence, having a practical and cost-effective tool to assess the degree of HSR failure is crucial in the context of obesity-related inflammation. This is particularly important because the expression of HSR components in PBMC inversely correlates with tissue pro-inflammatory states in chronic diseases, as evidenced in both animal and human studies [2,21,22,35,36].

Although C57BL/6J mice receiving an HFD can be accepted as a model of IR and diabetes, we observed that there were no significant differences in fasting blood glucose (Figure 10a), fasting insulin levels (Figure 10c), QUICK index (Figure 10e), HOMA (Figure 10g) or incremental area under the curve for oral GTT (Figure 12c) at the end of the study (22nd week). Furthermore, the ITT (Figure 13f and g) showed an impaired response to insulin only up to week 4 during the observational period in both NC and HFD groups. In addition, it is worth noting the limited effects of heat-induced HSP70 in control NC animals (Figure 9), which may partly reflect disrupted glycaemic status, including elevated fasting glycaemia after 10 weeks (*t*_1/2_ = 13.1 weeks) of *ad libitum* feeding (Figure 10) and insufficient physical activity, as previously reported [28]. Housing the animals within the TNZ probably suppressed expected metabolic changes in HFD animals, resulting in minimal metabolic alterations in both NC and HFD groups. Notably, prior research using a whole-body heat shock treatment [28] has shown transient HSP70 increases in skeletal muscle, liver and adipose tissue after acute heat treatment but a diminished response following chronic heat therapy (16 weeks) in HFD mice, consistent with findings in obese humans [24]. This absence of marked differences between control and heat-treated animals may be attributed to IFG observed between 6 and 13 weeks and QUICK indices indicating IR between 18 and 20 weeks (Figure 10). Despite this, both NC and HFD mice ended the 22-week period with similar obesity levels (Figure 8a). As HFDs effectively induce obesity-associated changes, the lack of more conspicuous differences in obesity levels (Figure 8) and heat-induced changes in NC mice (Figure 9) may stem from ambient housing temperatures. Unlike the present study, many rodents are housed below the TNZ (~21 °C), which affects metabolism and food intake [39–41,43]. At the T_a_ used in many studies (~21 °C), mice need to process significantly more energy per gramme of body mass than humans, even at BMR, resulting in greater metabolic heat production per gramme [39]. At sub-TNZ temperatures, HFD regimens yield pronounced metabolic effects. However, near the TNZ, these changes diminish, reflecting the strong genotype-phenotype link, limited activity, dietary choices and social interaction in animal models, which reduces their translational relevance to humans [42].

Actually, in commonly studied mice, *ad libitum* or HFDs, social isolation and restricted physical activity create a strong genotype-phenotype link, limiting variability in behaviour and diet compared with humans. However, when exposed to more human-like conditions, such as access to running wheels or cafeteria diets, obesity phenotypes are often mitigated or prevented [42]. These findings emphasise the critical role of exercise in preventing and treating obesity and suggest that limited food choices, akin to less palatable diets, may temporarily benefit humans by reducing obesity-related pathophysiology [42]. Taken together, these observations suggest that mice maintained within their TNZ and without any significant physical activity may experience weight gain in a trajectory much similar to that observed in HFD animals (Figure 8a), although their metabolic efficiency may be better as compared with HFD mice (Figure 8d). Considering that heat-induced HSP70 expression is tightly (and inversely) linked to the degree of weight gain [8], it was not surprising that heat-induced HSP70 had commenced to dissipate after 10 weeks of observation in control animals (Figure 9). To address these limitations, our laboratory is conducting comparative studies on diet-induced obesity in mice housed at TNZ (30 °C–34 °C), sub-TNZ (21 °C–22 °C) and supra-TNZ (35 °C), under Ethics Committee protocol #37,881.

Despite the above limitations, this is the first study to quantitatively assess the suppression of the anti-inflammatory HSR during obesity onset, concurrently evaluating deteriorating glycaemic status. Furthermore, control animals on an NC diet surpassed IR limits before the 22-week experimental period ends, equivalent to approximately 50 human years [46]. *Ad libitum* feeding and limited cage activity strongly contribute to this; sedentary animals show reduced intracellular HSP70 levels in adipose tissue, skeletal muscle and islets of Langerhans, reversed by exercise training [27] or chronic whole-body heat treatment [25], even under an HFD [25,27].

This study, a proof of concept, employed immunoblotting to detect ΔHSP70 variations. Currently, efforts are underway to address a limitation — correlating ΔHSP70 measured by ELISA and flow cytometry with hyperinsulinaemic/euglycaemic clamp data in obesity. This addresses a prior finding that diabetic individuals exhibit compromised HSR, assessed by *ex vivo* heat-induced eHSP70 release, reversed by resistance exercise training [21].

## Conclusion

**Figure 14:**
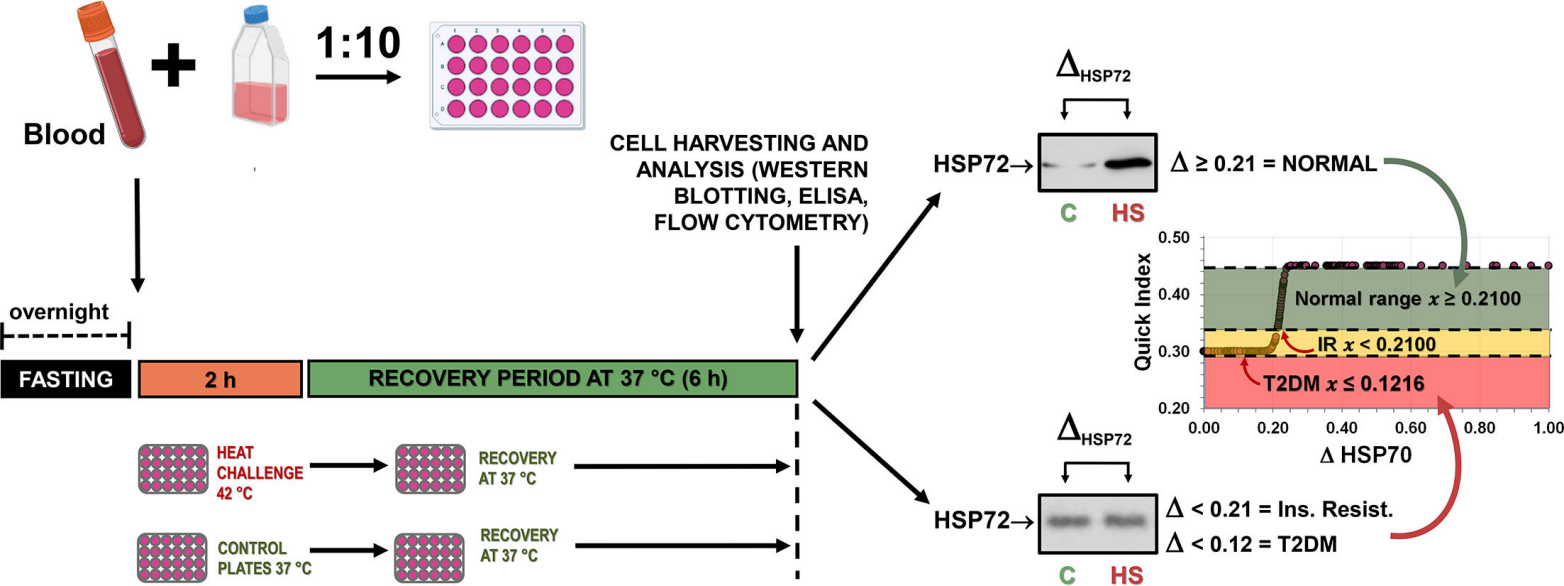
Summary figure. *Ex vivo* whole-blood heat shock challenge to assess organismal heat shock response (HSR). Heparinised blood samples are collected from overnight-fasted individuals and diluted 1:10 with a culture medium. The samples are incubated in a temperature-controlled water bath at 42.00 °C ± 0.01 °C for 2 hours, with parallel controls maintained at 37 °C for the same duration. Following incubation, cells from both groups are incubated for an additional 6 hours at 37 °C to allow robust accumulation of inducible HSP70 (HSP72), a marker of HSR capacity. The total experimental duration, from blood collection to the end of incubation, is 8 hours. HSP70 accumulation is assessed using Western blotting, ELISA or flow cytometry. The difference (ΔHSP72) between samples incubated at 42 °C and those at 37 °C correlates with the early onset of insulin resistance (IR) and type-2 diabetes mellitus (T2DM), even before clinical manifestations appear. QUICKI is the Quantitative Insulin Sensitivity Check Index. Adapted from reference [37]: Schroeder, H.T., et al.. 2024. Resolution of Inflammation in Chronic Disease via Restoration of the HSR. *Cell Stress Chaperones*. **29**(1):66–87. https://doi.org/10.1016/j.cstres.2024.01.005 (under an open access CC BY-NC-ND licence).

As a whole, this study provides a practical and cost-effective method for assessing HSR failure in the context of obesity-related inflammation, detectable well before clinical manifestations such as IFG, elevated fasting insulin, increased 2-hour post-glucose load glycaemia, or IR (please see the summary of the approach in Figure 14). It offers a valuable tool for the early detection of glycaemic progression in obese, insulin-resistant and type-2 diabetic patients. Notably, control animals on a standard diet, maintained near their TNZ, exceeded the IR threshold by week 22 — equivalent to approximately 50 human years. This finding is significant, as control diets are routinely used as benchmarks to evaluate dietary interventions in rodent models.

### Preclinical statement

As a preclinical study, the present work adheres to the NIH guidelines for reporting preclinical research (https://www.nih.gov/research-training/rigor-reproducibility/principles-guidelines-reporting-preclinical-research). In line with this, we have included, when appropriate, the following information: Detailed Statistics (appropriate section), Number of Replicates (in the Methods and figure legends), Randomisation (simple randomisation, https://www.graphpad.com/quickcalcs/randomN2/), Blinding (as stated in the methods, all manipulations of the animals and analyses were blindly performed by an investigator unaware of the groups) and Inclusion/Exclusion (after being randomly assigned, all the animals and samples were used). Although our present work is not dedicated to proposing any translational association between surrogate indices of glycaemic status between mice and humans, HOMA‑IR and QUICKI have been recently validated to be used in rodents, so we have performed correlations between our principal variable (ΔHSP70) and different indications of the glycaemic status of the animals, including HOMA‑IR and QUICKI, as comprehensively explained in the Methods section. Antibodies, their dilutions, equipment, buffers and other substances were appropriately furnished in the Methods. Statistical analyses were given in full in the appropriate section.

**Clinical perspectives**

- The anti-inflammatory heat shock response (HSR) is progressively suppressed in chronic diseases of an inflammatory nature. HSR failure is commonly observed in obesity, insulin resistance, diabetes and CVD.
- We assessed the HSR via heat-challenged whole-blood samples from high-fat diet mice and discovered that ΔHSP70 expression = (HSP70 at 42 °C) **–** (HSP70 at 37 °C) fits a five-parameter logistic (5PL) regression with the progression of insulin resistance and predicts the onset of type-2 diabetes.
- ΔHSP70 expression assessed by heat-challenging blood samples at 42 °C offers a cost-effective tool for clinical evaluation of the progression of the HSR in obesity, insulin resistance and type-2 diabetes before any clinical manifestation.

## Supplementary material

Supplementary Figures

## Data Availability

Original data are available from the corresponding author upon request.

## Open Access

This article has been published open access under our Subscribe to Open programme, made possible through the support of our subscribing institutions, learn more here: https://portlandpress.com/pages/open_access_options_and_prices#conditional

## Abbreviations

AMPK: 5’-adenosine monophosphate-activated protein kinase
ARRIVE: Animal Research: Reporting of In Vivo Experiments
CVD: Cardiovascular Disease
ER: endoplasmic reticulum
GAPDH: Glyceraldehyde-3-phosphate dehydrogenase
HFD: high-fat diet
HOMA-IR: Homeostasis Model Assessment of Insulin Resistance
HSF1: heat shock transcription factor-1
HSP70: the 70 kDa family of heat shock proteins
HSP: heat shock protein
HSR: heat shock response
HuR: human antigen R
IFG: impaired fasting glycaemia
IGT: impaired glucose tolerance
JNK: c-Jun N-terminal kinases
NC: standard normal chow
NLRP3: NOD-like receptor pyrin domain-containing protein-3 inflammasome
PBL: peripheral blood leukocytes
PBMC: peripheral blood mononuclear cells
PGC-1α: peroxisome proliferator-activated receptor-γ coactivator‑1α
QQ plot: Quantile-Quantile plot
QUICKI: Quantitative Insulin Sensitivity Check Index
RIPA: Radio-Immunoprecipitation Assay
SASP: Senescence-Associated Secretory Phenotype
SIRT1: NAD^+^-dependent deacetylase sirtuin-1
TLR: toll-like receptor
UPR: unfolded protein response
USP: United States Pharmacopoeia
a.k.a. ELAV-1: for Embryonic Lethal, Abnormal Vision, Drosophila, Homolog-Like protein-1
eHSP70: extracellular HSP70
ipITT: intraperitoneal Insulin Tolerance Test
oGTT: oral Glucose Tolerance Test
