# Supplementary figures and images for: Early detection and progression of insulin resistance revealed by impaired organismal anti-inflammatory heat shock response during ex vivo whole-blood heat challenge
Source: Clin Sci (Lond). 2025 Jan 23;139(2):85–113. doi: 10.1042/CS20243515 (PMC12204015; doi:10.1042/CS20243515)

a)

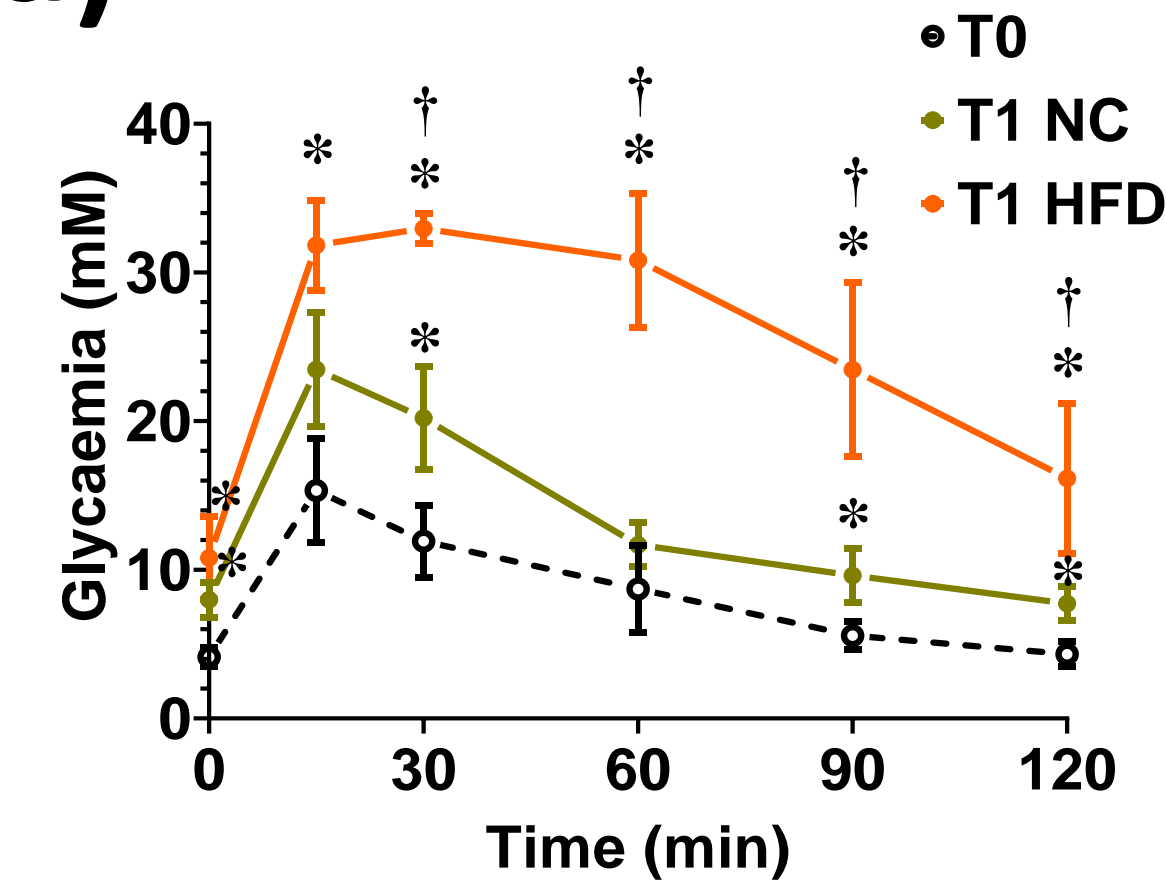

b)

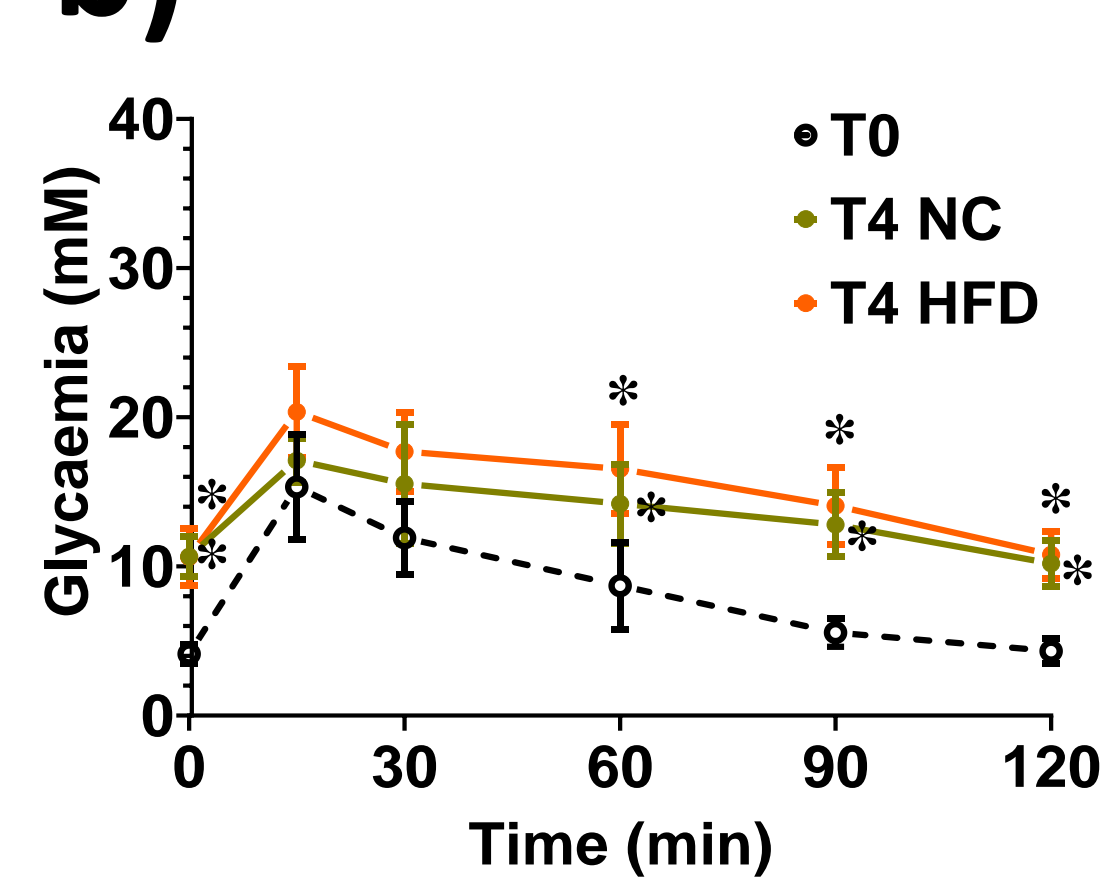

c)

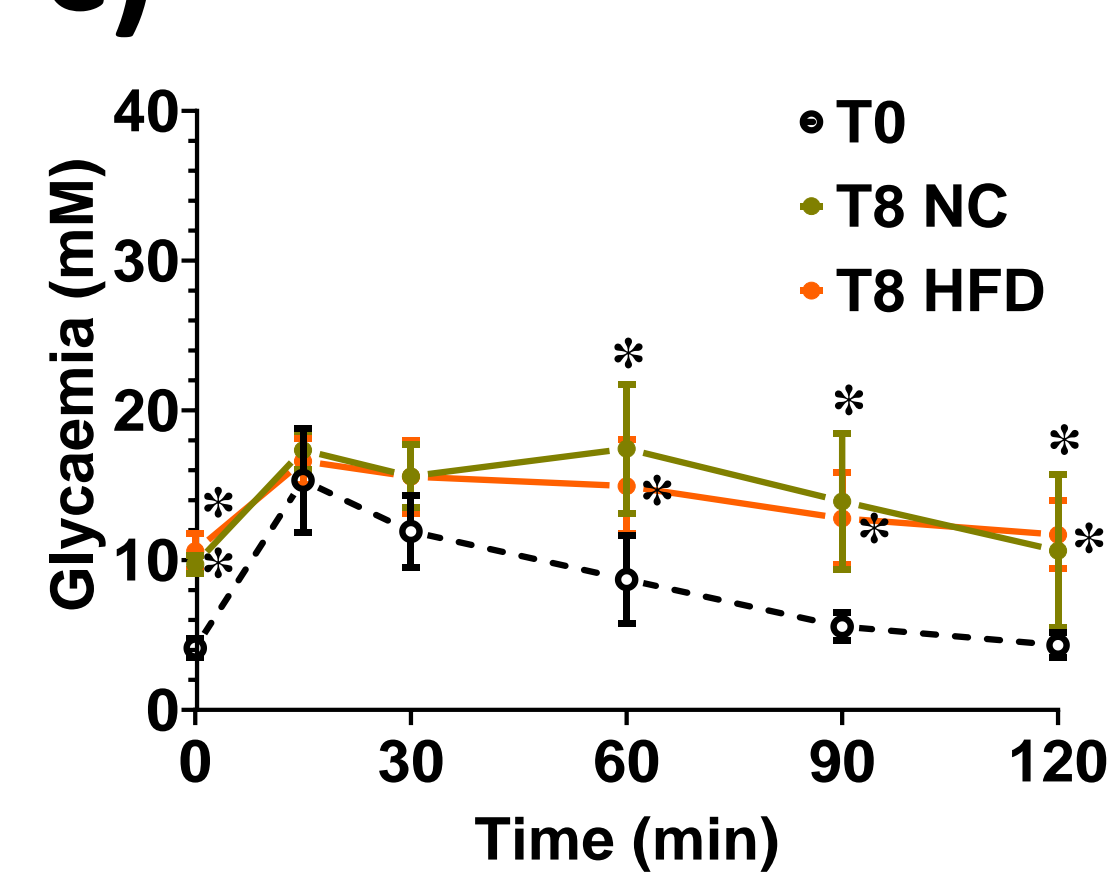

d)

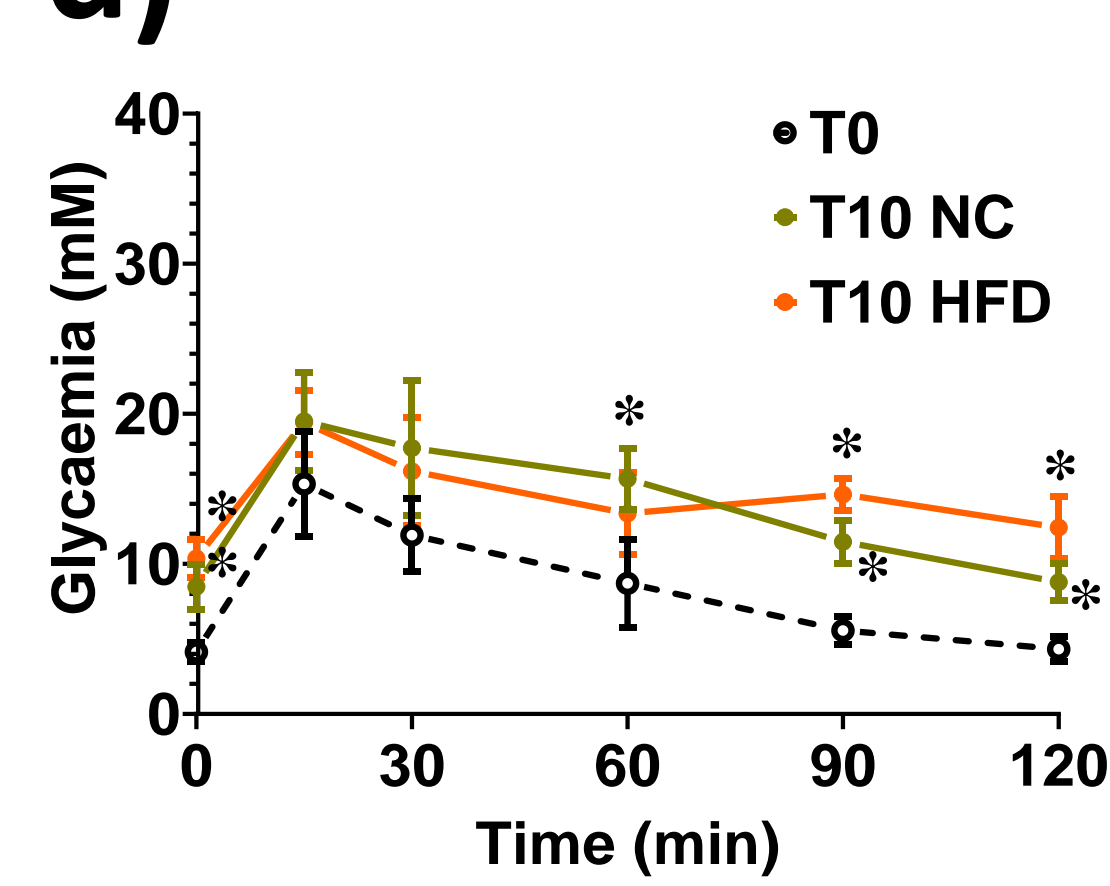

e)

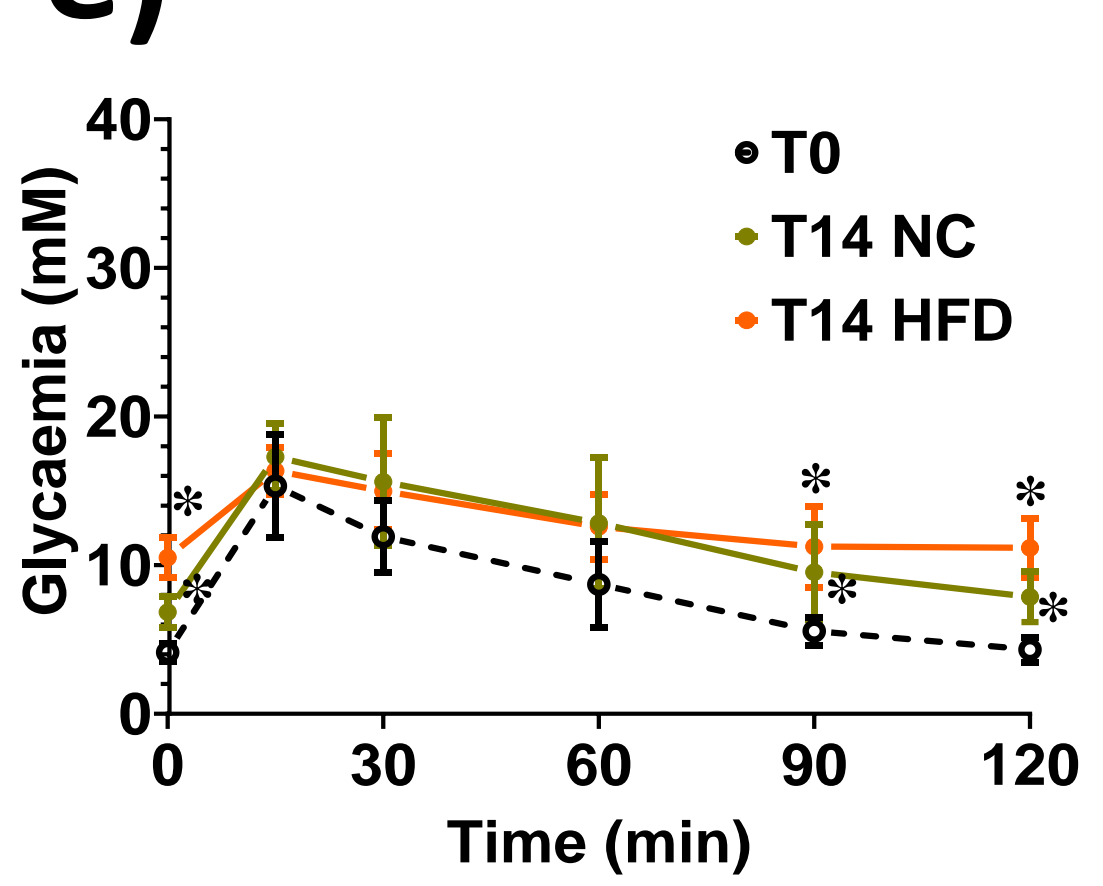

f)

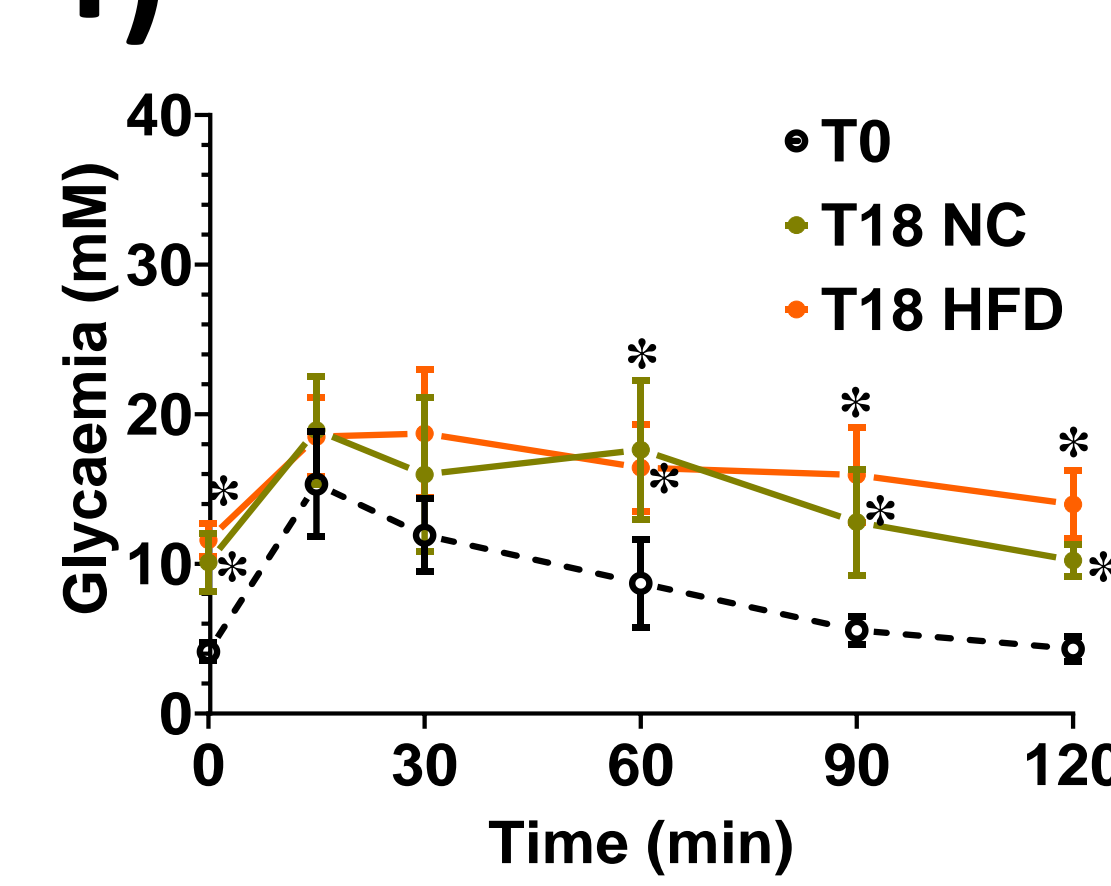

g)

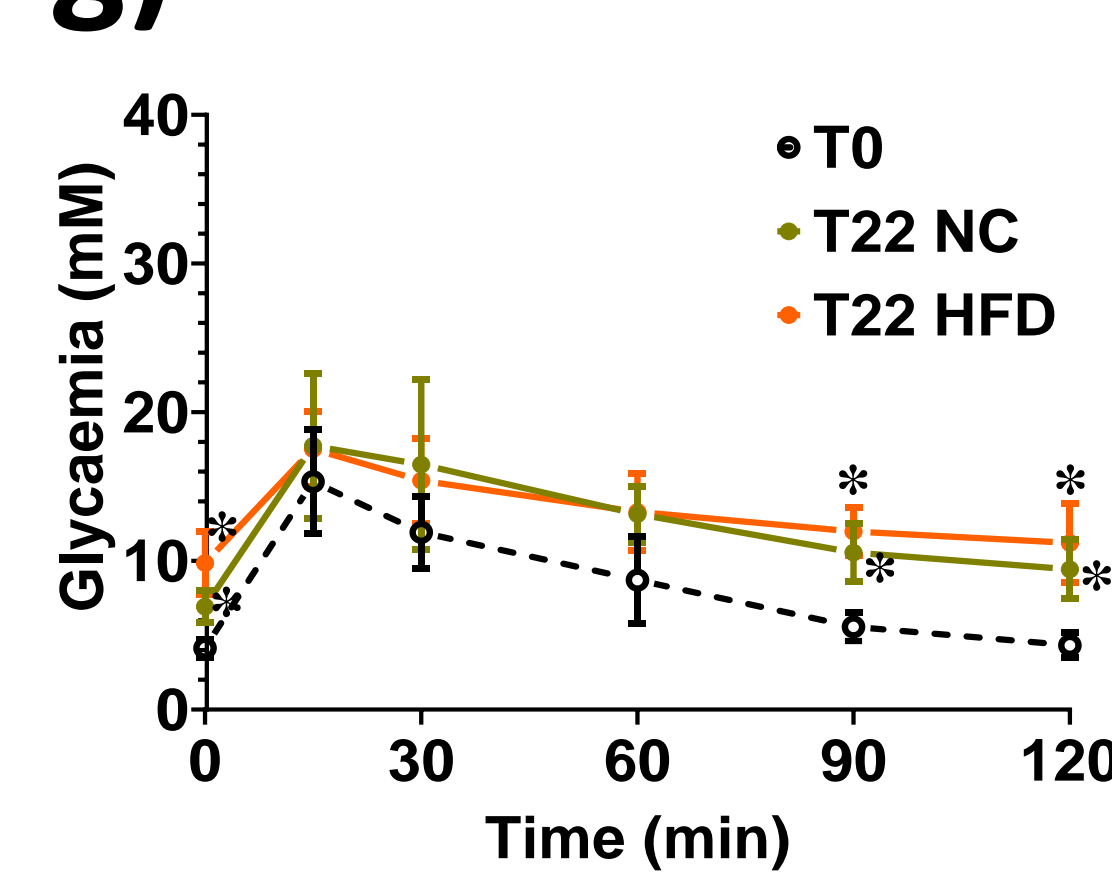

h)

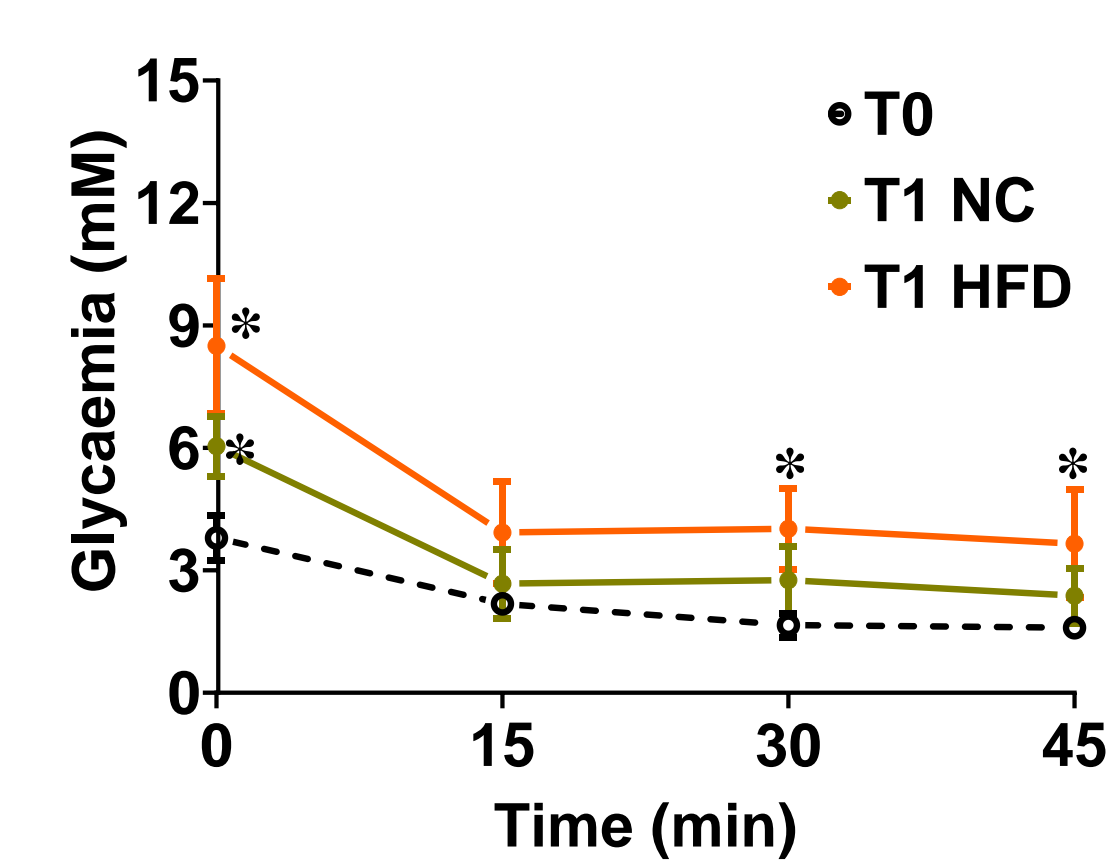

i)

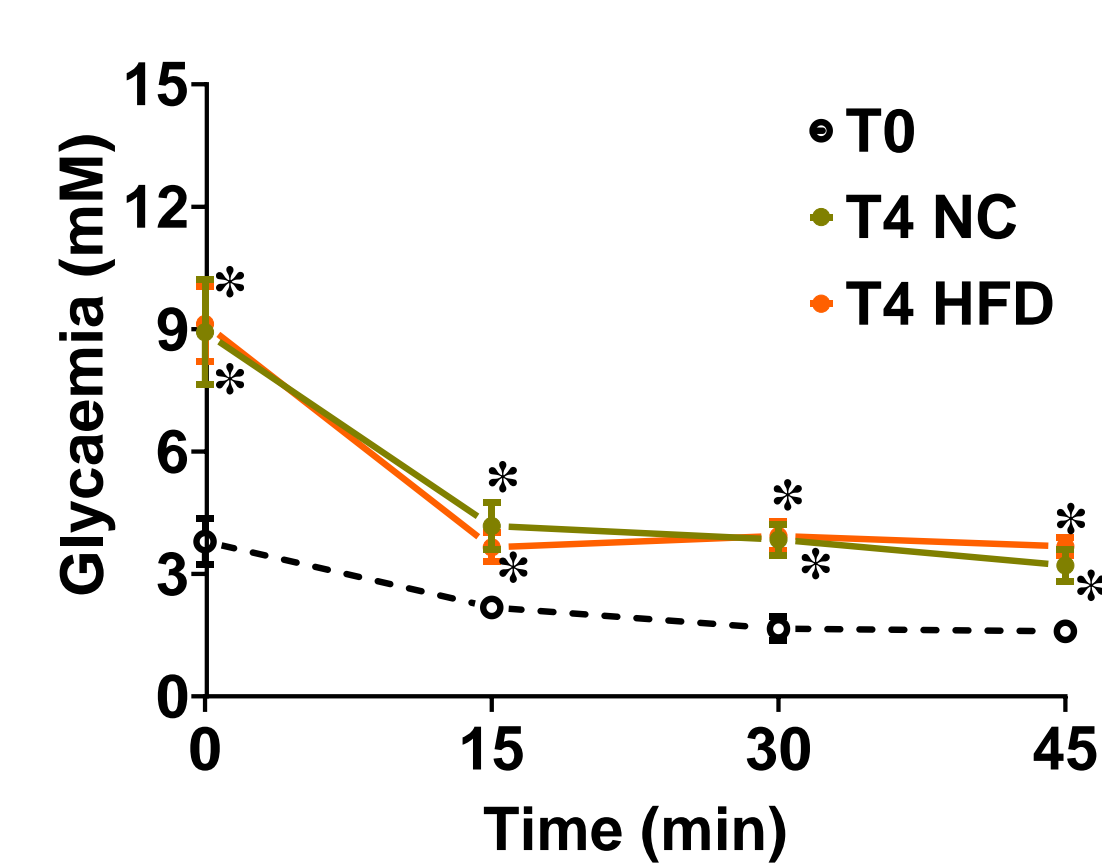

j)

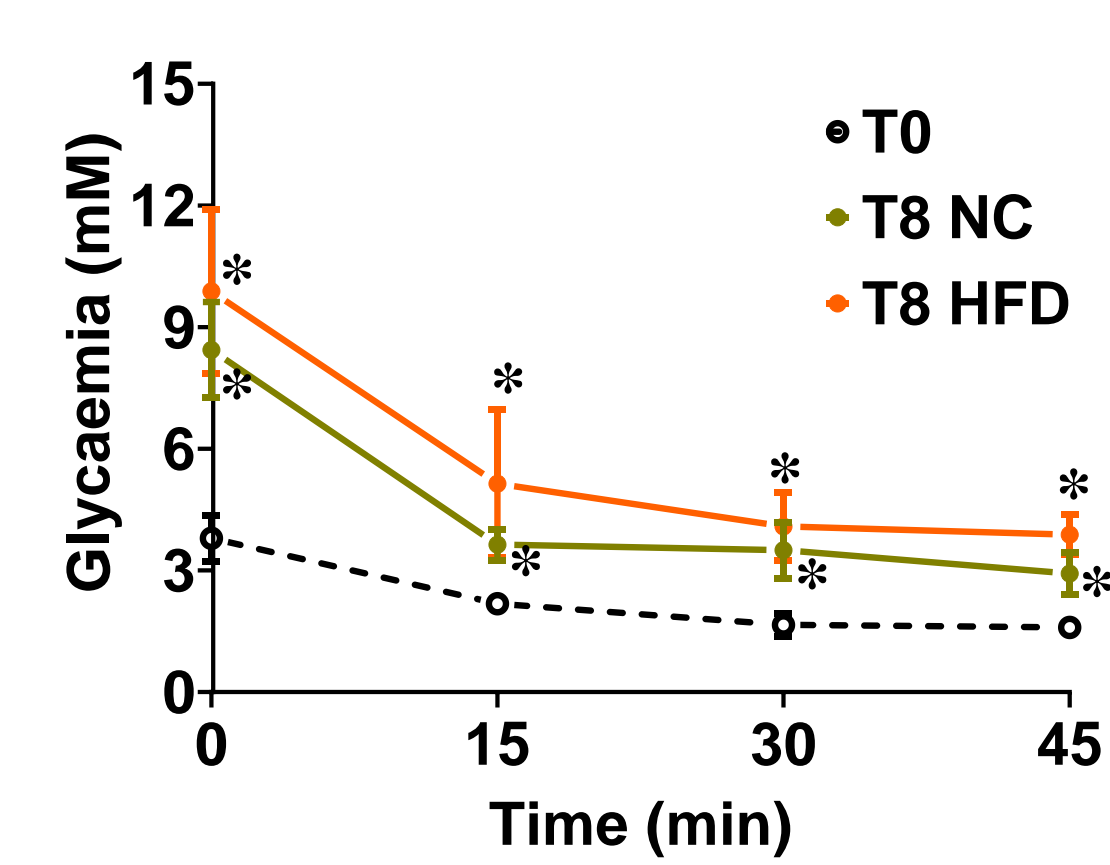

k)

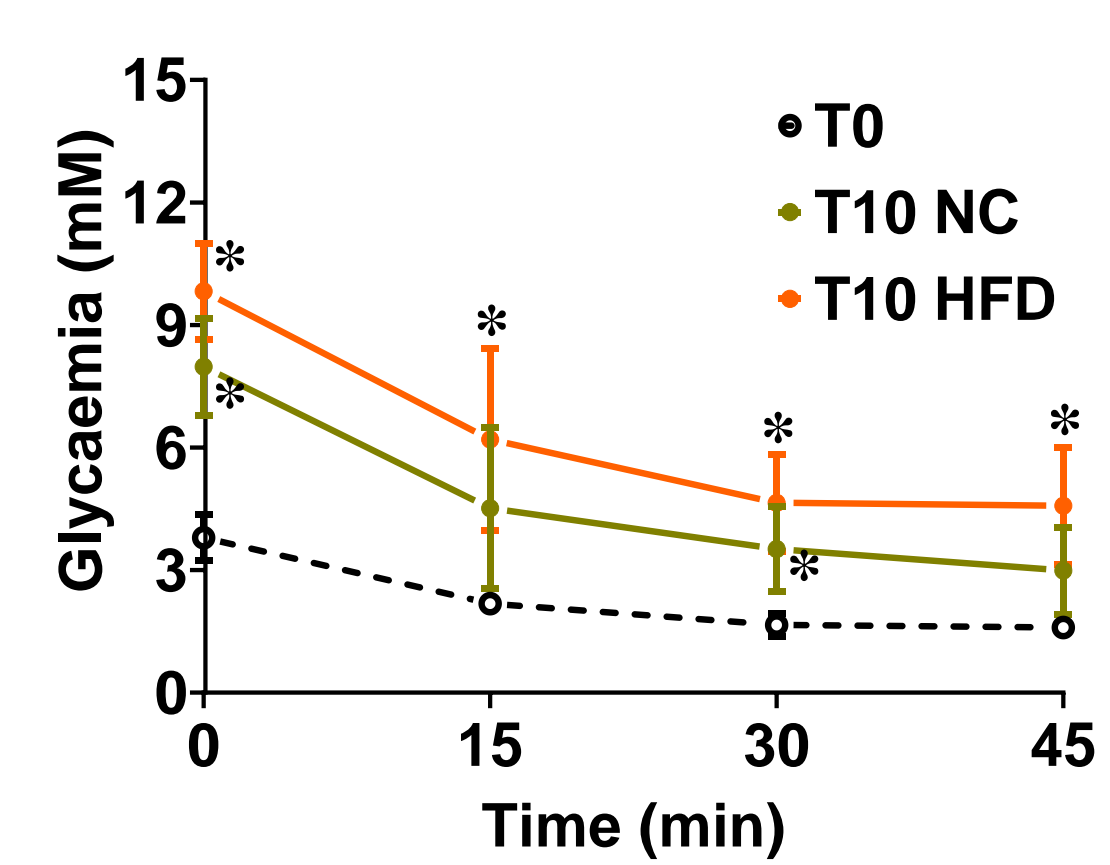

l)

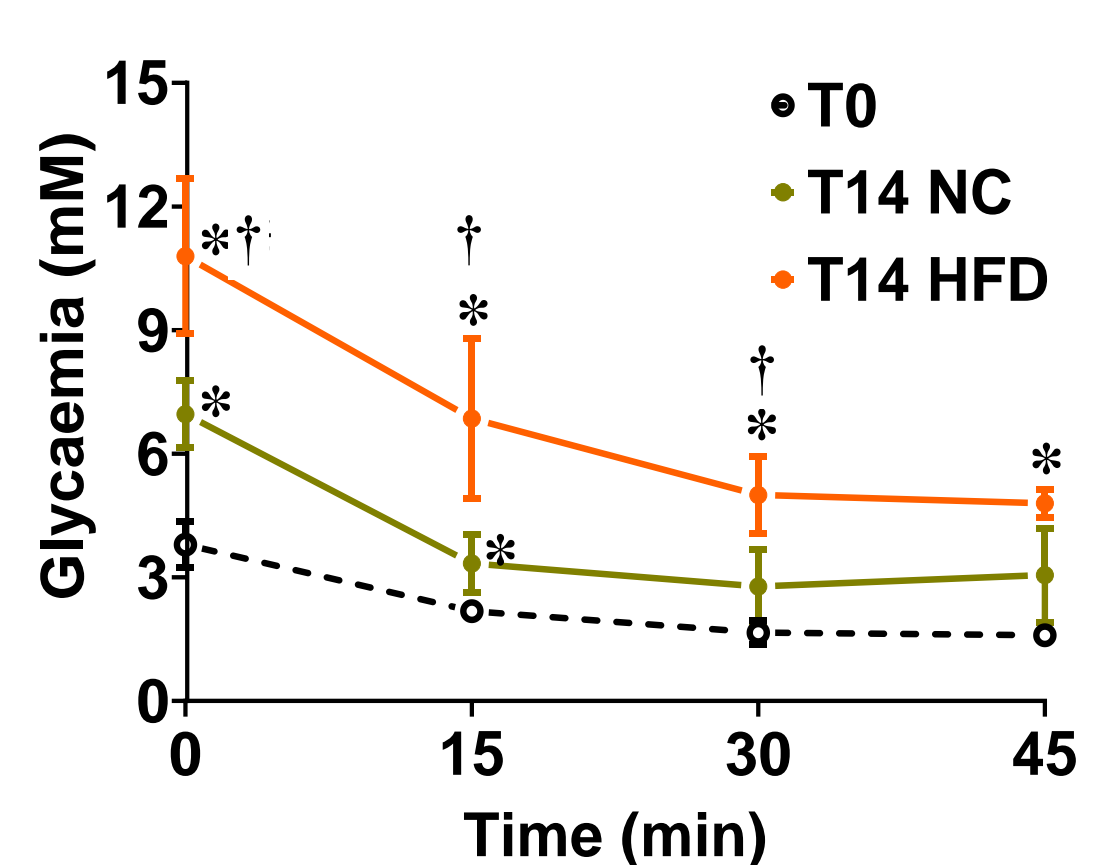

m)

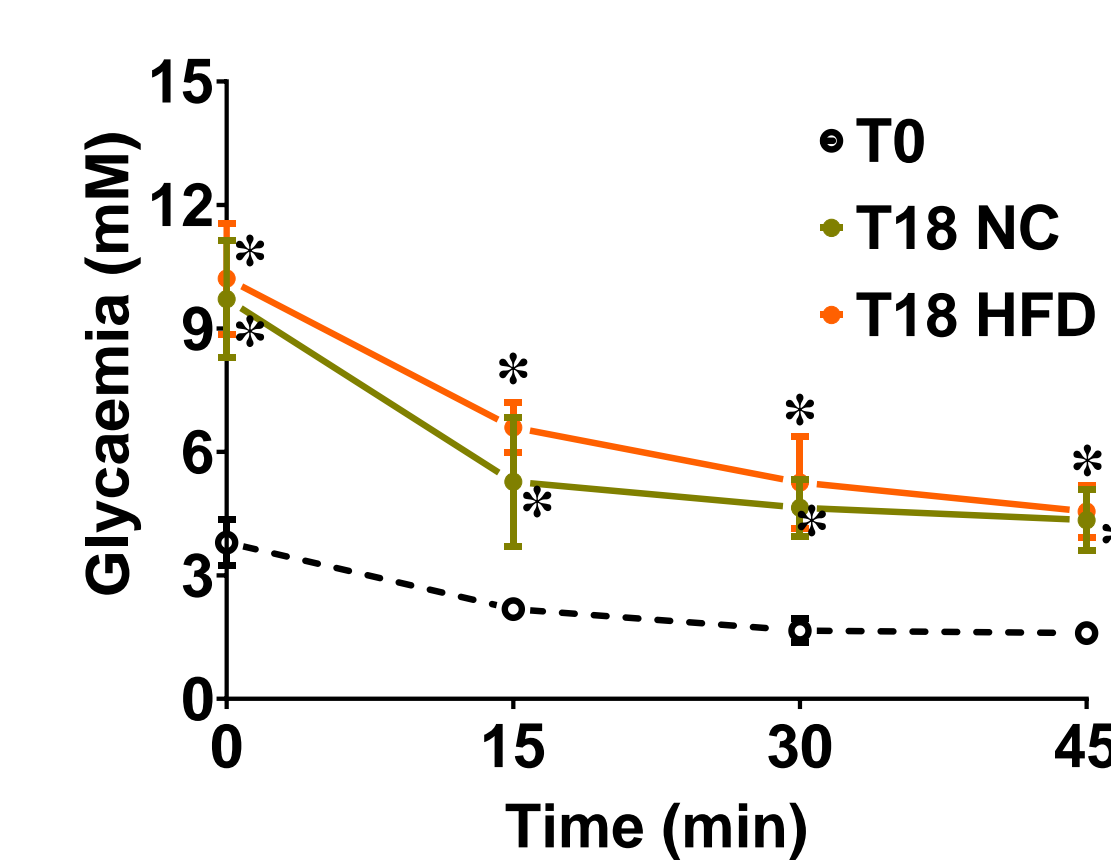

n)

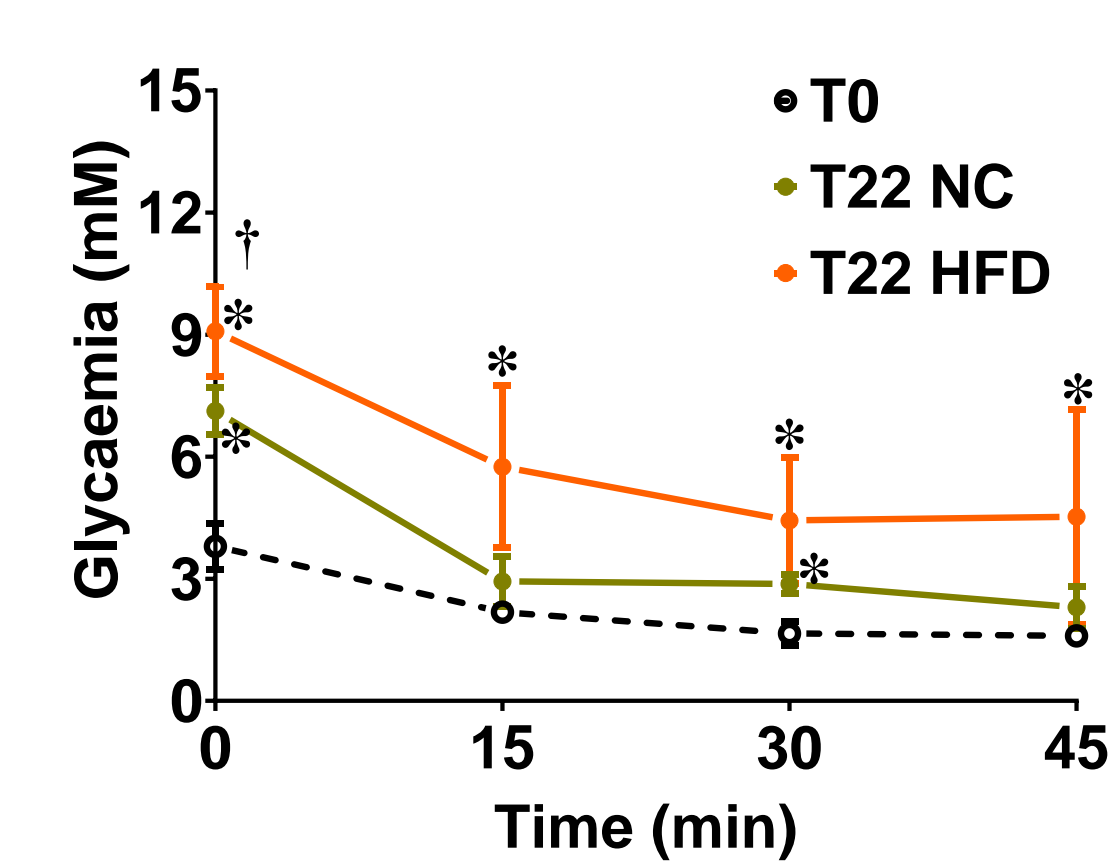

Supplement: Supplementary Figures [file CS-139-02-CS20243515-s001.pdf]
